# Supplementary material for: Directed-evolution of translation system for efficient unnatural amino acids incorporation and generalizable synthetic auxotroph construction
Source: Nat Commun. 2021 Dec 2;12:7039. doi: 10.1038/s41467-021-27399-x (PMC8639764; doi:10.1038/s41467-021-27399-x)
Supplement: Supplementary file 1 — Supplementary Information [file 41467_2021_27399_MOESM1_ESM.pdf]

Supplementary Information for:

Zhao et al.

Directed-evolution of translation system for efficient unnatural amino acids incorporation and generalizable synthetic auxotroph construction

Supplementary Tables, Figures and Sequences

Supplementary Table 1. The chPheRS mutation table

Supplementary Figure 1. The amino acids used in this study

Supplementary Figure 2. The schematic of directed evolution process for the chPheT and chPheRS

Supplementary Figure 3. Amber suppression efficiency in mammalian cells.

Supplementary Figure 4. Western blot analysis of full-length and truncation GFP expression

Supplementary Figure 5. Single-site incorporation of AzF at various sites of GFP using 12D4-AzFRS-2

Supplementary Figure 6. Single-site incorporation of AzF at various sites of GFP using AzFRS-2

Supplementary Figure 7. Characterization of chromosomally integrated 12D4-AzFRS-2

Supplementary Figure 8. Kinetic analysis of GFP production by chPheRSs

Supplementary Figure 9. The fitness of cell expressed with the evolved pair

Supplementary Figure 10. The incorporation fidelity of AzF at various proteins

Supplementary Figure 11. The incorporation fidelity of multi-site AzFs

Supplementary Figure 12. Amber suppression efficiency of the AzF system and the BTA system

Supplementary Figure 13. The comparison of AzF incorporation systems

Supplementary Figure 14. Western blot analysis of three-site AzFs incorporation at essential proteins

Supplementary Figure 15. Verification of knockout clones

Supplementary Figure 16. Escape frequencies of auxotrophic strains

Supplementary Sequence 1. The sequences of DNA fragments used to construct UAA-dependent synthetic auxotrophs

Supplementary Sequence 2. The DNA sequences of OTS integration cassette

Supplementary Sequence 3. The sequences of promoter in this study

Supplementary Sequence 4. The DNA sequences of chPheTs obtained from selection

Supplementary Sequence 5. The DNA and protein sequences of the evolved chPheRS

Supplementary Sequence 6. The plasmid maps and sequences

Supplementary Sequence 7. The DNA sequences of proteins for amber suppression assay

Supplementary Sequence 8. The table of PCR primers for DNA constructions and mutations

Supplementary Table 1. The chPheRS mutation table. The mutation of chPheRS variants used in this study are shown.

chPheRS	UAA	Mutation
NapARS	2-Naphthyl-Ala	T467G, A507G
AcFRS	4-Acetyl-Phe	T467G, A507G
AzFRS-1	4-Azido-Phe	T467G, A507G
6MWRS	6-Methyl-Trp	E391D, T467G, A507G
7MWRS	7-Methyl-Trp	F464V, T467G, A507G
BTARS	3-Benzothienyl-Ala	F464V, T467G, A507G
AzFRS-2	4-Azido-Phe	F464I, T467G, A507G
12D4-AzFRS-2	4-Azido-Phe	K290L, Y294N, R300S, F301S, S333C, S397T, M490L, L455P, F464I, T467G, A507G

Supplementary Figures

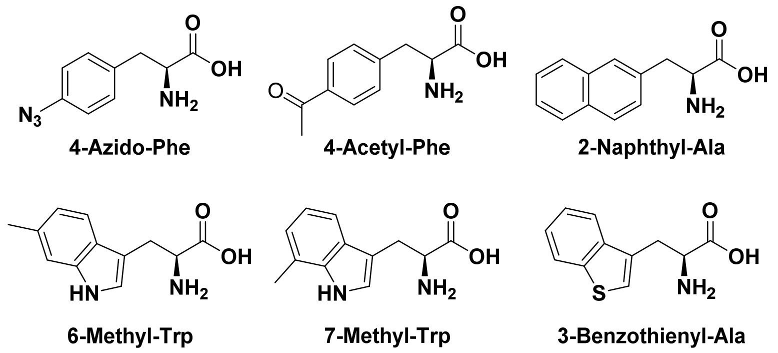

Supplementary Figure 1. The amino acids used in this study. The chemical structures and common names of amino acids used in this study are shown.

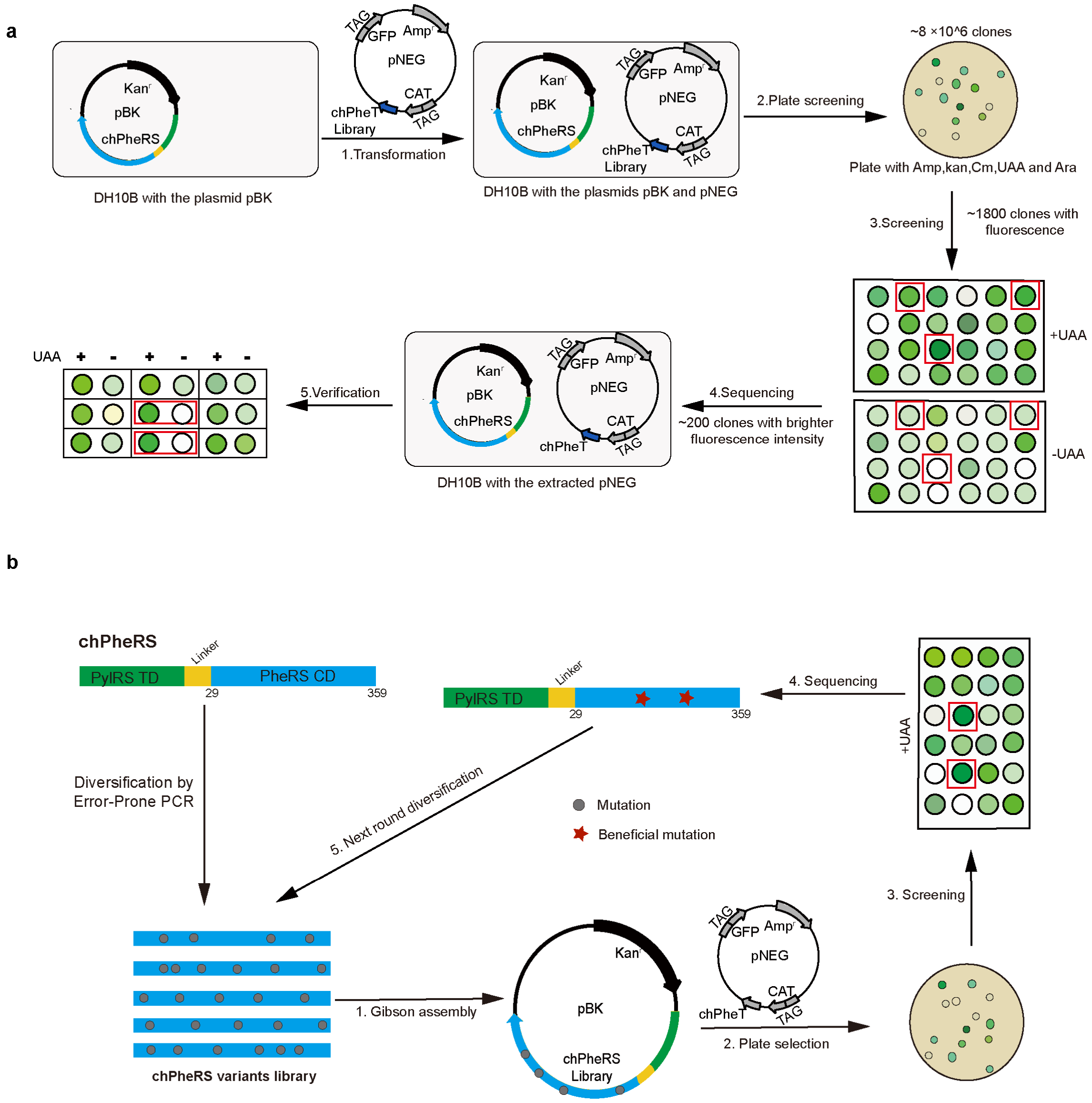

Supplementary Figure 2. The schematic of directed evolution process for the chPheT and chPheRS. (a) The direction evolution of the chPheT. The tRNA library was subjected to one round of positive selection to identify orthogonal chPheTs that showed improved amber suppression efficiency compared to the progenitor chPheT. (b) The direction evolution of the chPheRS. Four rounds of iterative selection were performed to identify orthogonal chPheRSs that showed improved amber suppression efficiency compared to the progenitor chPheRS.

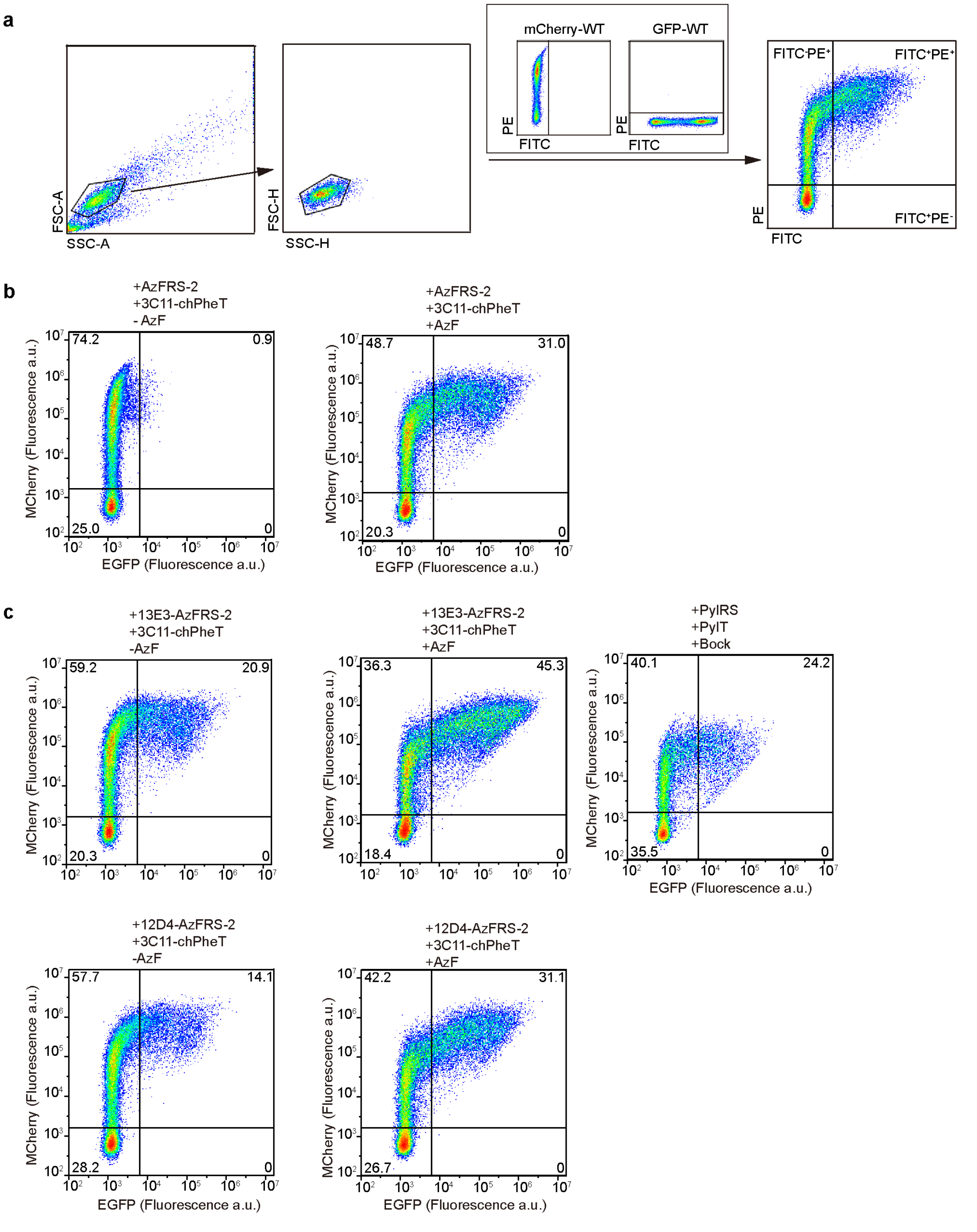

Supplementary Figure 3. Amber suppression efficiency in mammalian cells. (a) Gating strategy for transfected cells in FlowJo. HEK 293T cells were used to set appropriate forward scatter (FSC) and side scatter (SSC) gains. EGFP-WT cells and mCherry-WT cells were used as positive control to set FITC and PE gate, which was highlighted by black boxes. The fluorescence intensity values for the individual mCherry-

positive cells are exported for downstream analysis. (b) Flow cytometry analysis of amber suppression efficiency in HEK 293T cells transfected with the AzFRS-2 and the 3C11-chPheT. (c) Flow cytometry analysis of amber suppression efficiency in HEK 293T cells transfected with the indicated orthogonal translation systems.

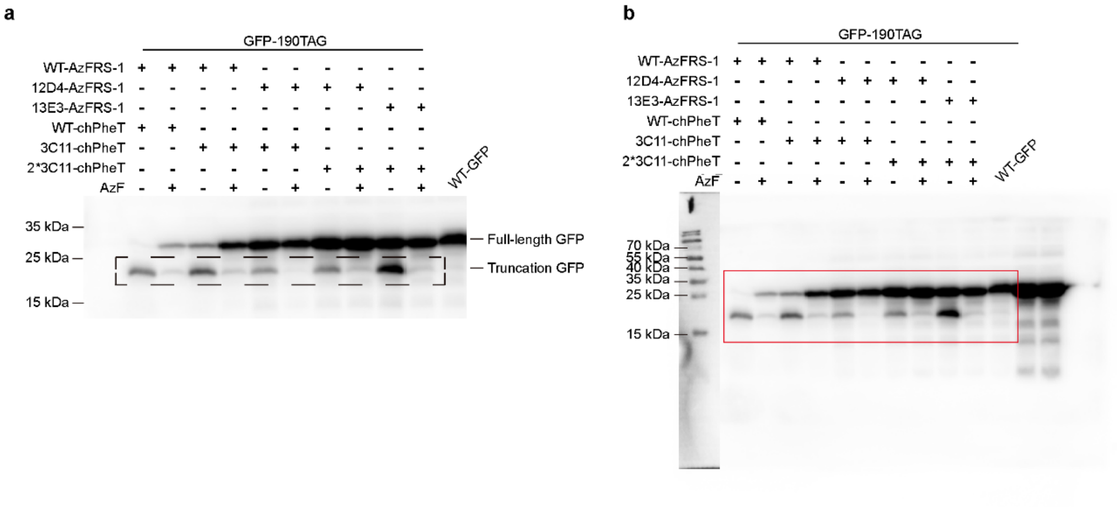

Supplementary Figure 4. Western blot analysis of full-length and truncation GFP expression. (a) The indicated orthogonal translation systems and the GFP-190TAG were co-transformed into DH10B to assay the expression of full-length and truncation GFP expression. The truncated protein is highlighted by a black dashed-box. (b) The full blots showed in Supplementary Figure 4a, the region in Supplementary Figure 4a was highlighted by a red solid-box. The experiment in the figure was repeated twice with similar results. Source data are provided as a Source Data file.

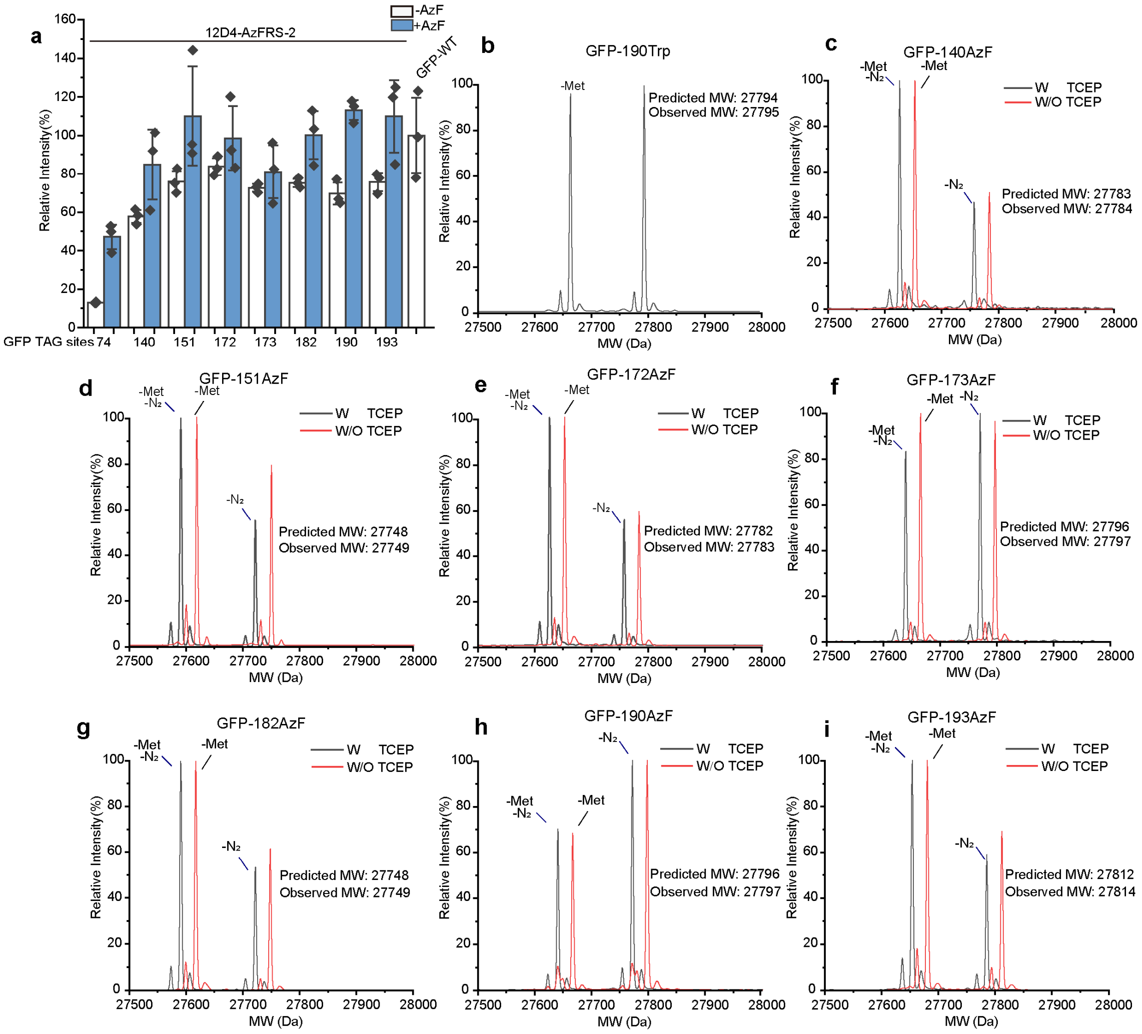

Supplementary Figure 5. Single-site incorporation of AzF at various sites of GFP using 12D4-AzFRS-2. (a) Amber suppression efficiency of GFP carrying amber codon at the indicated position by the 12D4-AzFRS-2 system was examined in the presence or absence of 1 mM AzF. Error bars represents ±standard error of the mean from three biologically independent experiments. (b) Mass spectrometry characterization of GFP-190Trp. (c-i) Mass spectrometry characterization of the fidelity of AzF incorporation into various sites of GFP using the 12D4-AzFRS-2/3C11-chPheT pair. The fidelity of AzF incorporation was further evaluated by TCEP reduction. The MW of these GFP variants before (red line) and after (black line) TCEP treatment were shown. In each spectrum, N′-Met cleaved peaks were also detected. Source data are provided as a Source Data file.

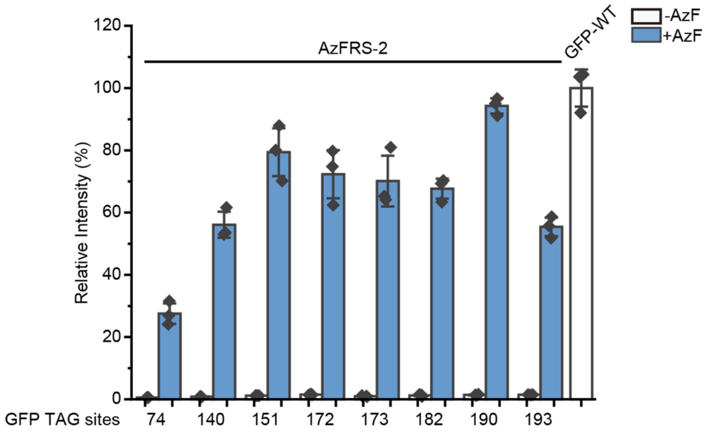

Supplementary Figure 6. Single-site incorporation of AzF at various sites of GFP using AzFRS-2. Amber suppression efficiency of GFP carrying amber codon at the indicated position by the AzFRS-2/3C11-chPheT pair was examined in the presence or absence of 1 mM AzF. Error bars represents ±standard error of the mean from three biologically independent experiments. Source data are provided as a Source Data file.

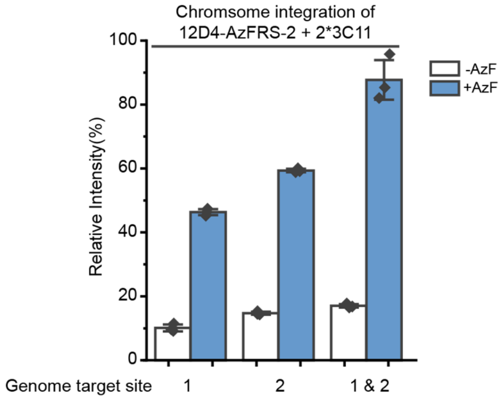

Supplementary Figure 7. Characterization of chromosomally integrated 12D4-AzFRS-2. Amber suppression efficiency of GFP in DH10B chromosomally integrated 12D4-AzFRS-2 under the control of *oxb20* promoter was assayed in the presence or absence of 1 mM AzF (Site 1: chromosome position 17231-17232; Site 2: chromosome position 2048790-2048791). Error bars represents ±standard error of the mean from three biologically independent experiments. Source data are provided as a Source Data file.

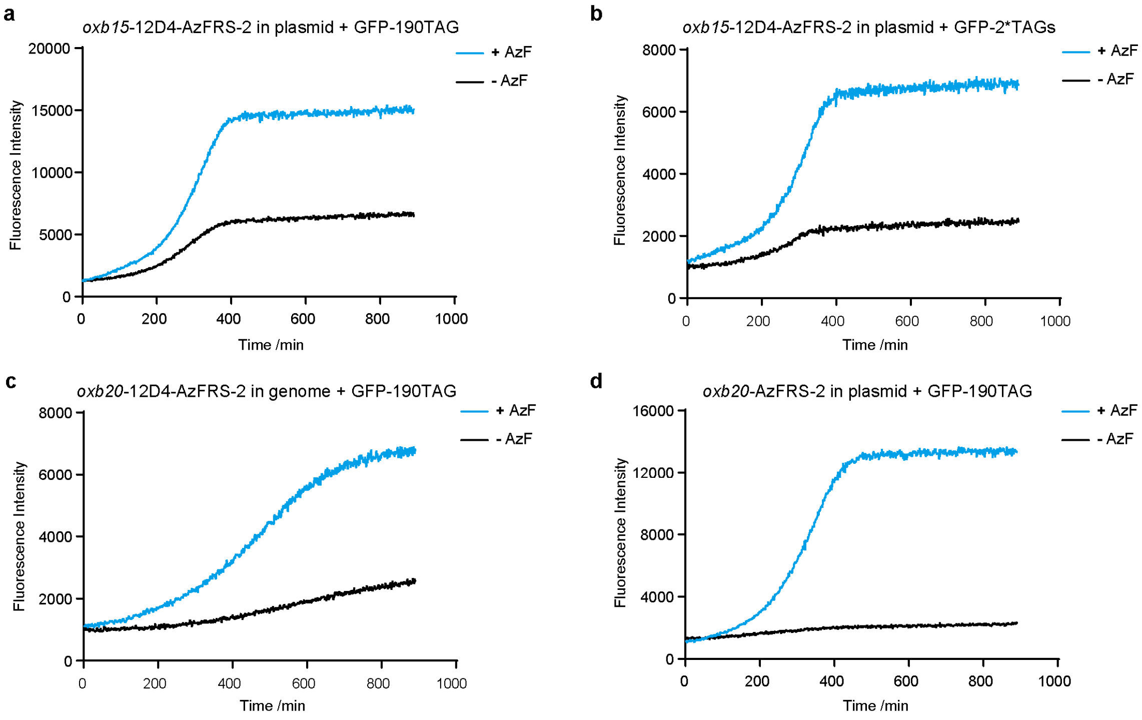

Supplementary Figure 8. Kinetic analysis of GFP production by chPheRSs. Time course analysis of GFP-190TAG or GFP-2*TAGs (151TAG and 190TAG) expression by the indicated systems in the presence or absence of 1 mM AzF. (a) plasmid-based overexpression of 12D4-AzFRS-2 with *oxb15* promoter, (b) plasmid-based overexpression of 12D4-AzFRS-2 with *oxb15* promoter, (c) genome-integrated overexpression of 12D4-AzFRS-2 with *oxb20* promoter, (d) plasmid-based overexpression of AzFRS-2 with *oxb20* promoter. *Oxb15* promoter is a strongly synthetic constitutive promoter which is slightly weaker than *oxb20* promoter. The experiments in the figure were repeated twice with similar results. Source data are provided as a Source Data file.

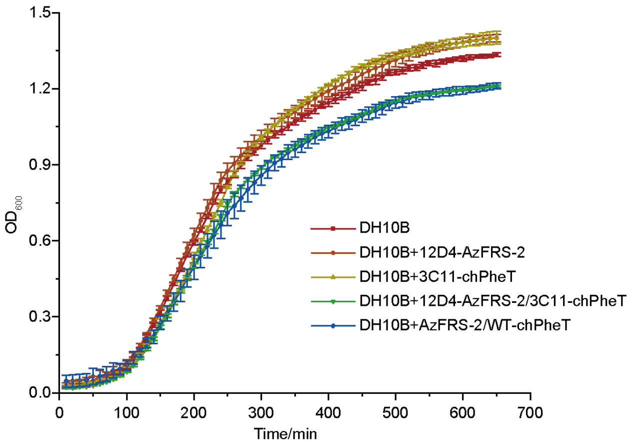

Supplementary Figure 9. The fitness of cell expressed with the evolved pair. Growth curve of various DH10B strains with or without the indicated chimeric Phe systems. Error bars represents ± standard error of the mean from three biologically independent experiments. Source data are provided as a Source Data file.

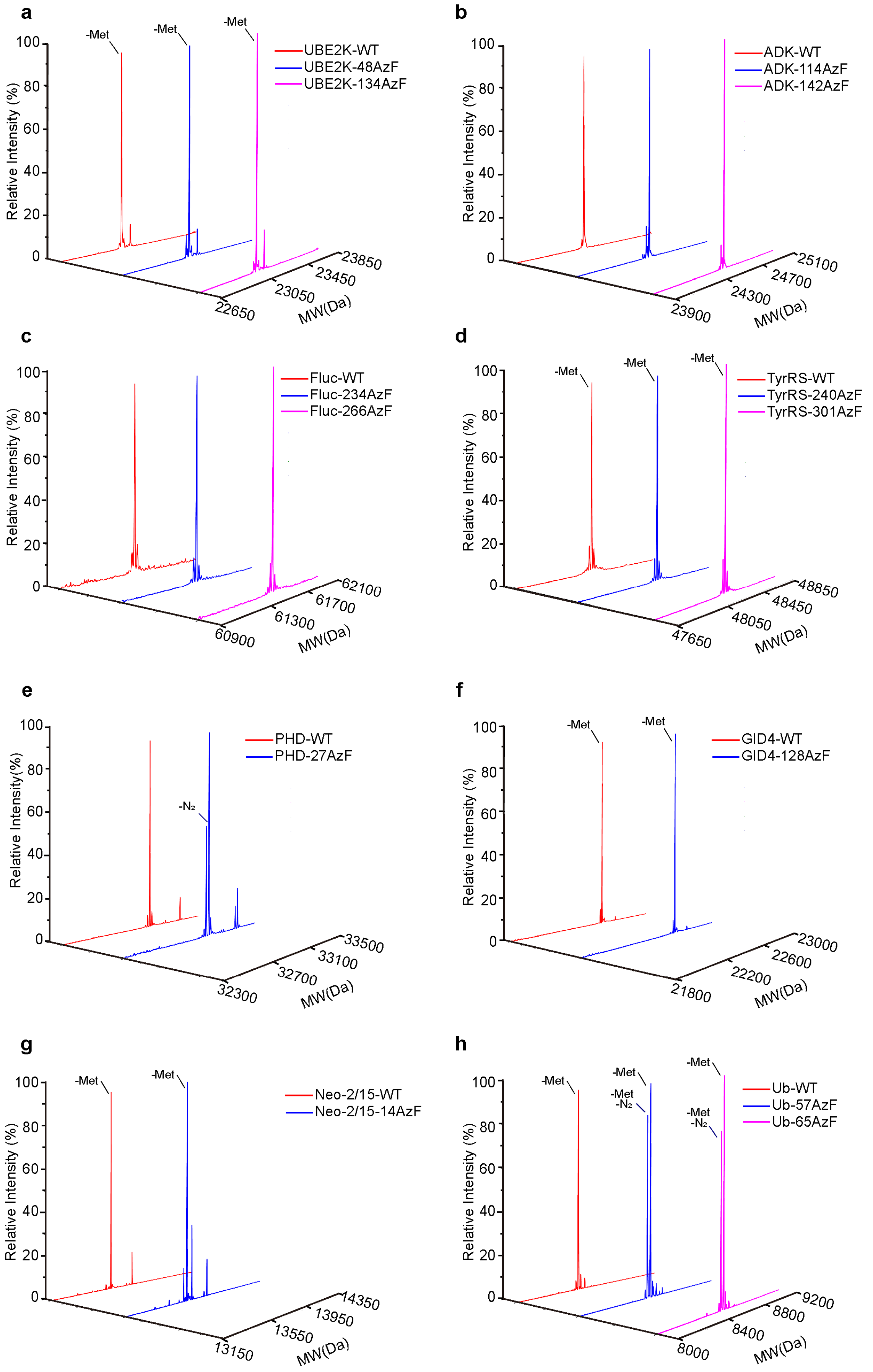

a
-Met
-Met
-Met
UBE2K-WT
UBE2K-48AzF
UBE2K-134AzF
Relative Intensity (%)
100
80
60
40
20
0
22650
23050
23450
23850
MW(Da)
b
ADK-WT
ADK-114AzF
ADK-142AzF
Relative Intensity (%)
23900
24300
24700
25100
MW(Da)
c
Fluc-WT
Fluc-234AzF
Fluc-266AzF
Relative Intensity (%)
60900
61300
61700
62100
MW(Da)
d
-Met
-Met
-Met
TyrRS-WT
TyrRS-240AzF
TyrRS-301AzF
Relative Intensity (%)
47650
48050
48450
48850
MW(Da)
e
PHD-WT
PHD-27AzF
-N₂
Relative Intensity(%)
32300
32700
33100
33500
MW(Da)
f
-Met
-Met
GID4-WT
GID4-128AzF
Relative Intensity (%)
21800
22200
22600
23000
MW(Da)
g
-Met
-Met
Neo-2/15-WT
Neo-2/15-14AzF
Relative Intensity (%)
13150
13550
13950
14350
MW(Da)
h
-Met
-Met
-Met -N₂
-Met
-Met -N₂
Ub-WT
Ub-57AzF
Ub-65AzF
Relative Intensity (%)
8000
8400
8800
9200
MW(Da)

Supplementary Figure 10. The incorporation fidelity of AzF at various proteins. Mass spectrometry characterization of the fidelity of AzF incorporation into UBE2K (a) the expected MW (-Met) from left to right are 23098, 23171, and 23123 Da, and the observed MW (-Met) are 23099, 23172, and 23124 Da; into ADK (b) the expected MW from left to right are 24409, 24468, and 24498 Da, and the observed MW are 24410, 24469, and 24499 Da; into firefly luciferase (Fluc) (c) the expected MW from left to right are 61468, 61492 and 61540 Da, and the observed MW are 61468, 61494 and 61542 Da; into TyrRS (d) the expected MW (-Met) from left to right are 48219, 48278 and 48244 Da, and the observed MW (-Met) are 48222, 48280 and 48247 Da; into PHD (e) the expected MW from left to right are 32986 and 32988 Da, and the observed MW are 32987 and 32989 Da; into GID4 (f) the expected MW(-Met) from left to right are 22532 and 22573 Da, and the observed MW(-Met) are 22533 and 22574 Da; into Neo-2/15 (g) the expected MW(-Met) from left to right are 13578 and 13603 Da, and the observed MW(-Met) are 13579 and 13604 Da; into Ub (h) the expected MW(-Met) from left to right are 8434, 8535, and 8535 Da, and the observed MW(-Met) are 8454, 8555, and 8555 Da. When AzF was incorporated into PHD and Ub, azide reduction peaks are also detected. The azide reduction is likely caused by using reducing reagent during protein purification. Although the expected MW and the observed MW of wild-type Ub is different, excellent AzF incorporation on Ub is detected. The detailed sequences of these proteins are shown in Supplementary Sequences. Source data are provided as a Source Data file.

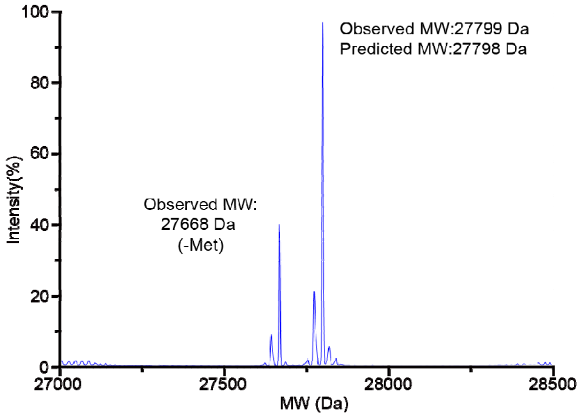

Supplementary Figure 11. The incorporation fidelity of multi-site AzFs. Mass spectrometry characterization of the fidelity of three-site AzFs incorporation into GFP with the 12D4-AzFRS-2/3C11-chPheT pair. Source data are provided as a Source Data file.

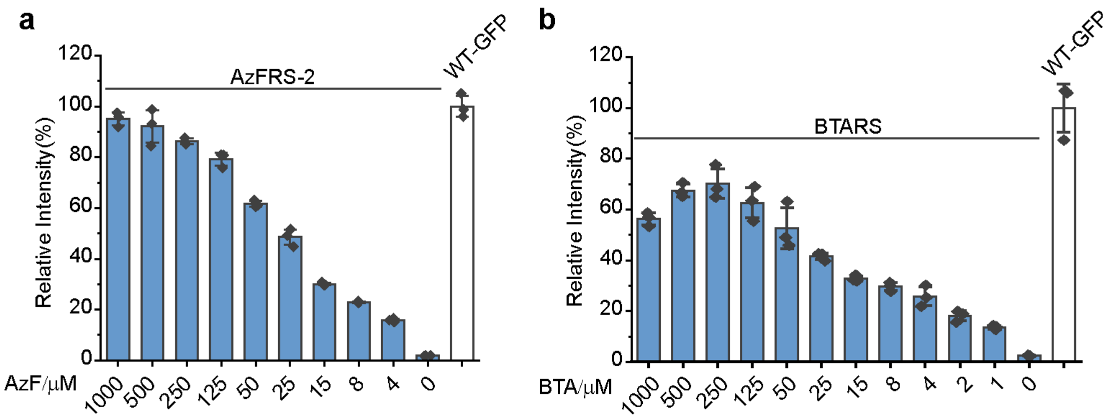

Supplementary Figure 12. **Amber suppression efficiency of the AzF system and the BTA system.** Amber suppression efficiency of the AzF system (a) and the BTA system (b) is tested in the presence of indicated concentration of AzF or BTA. Error bars represents ±standard error of the mean from three biologically independent experiments. Source data are provided as a Source Data file.

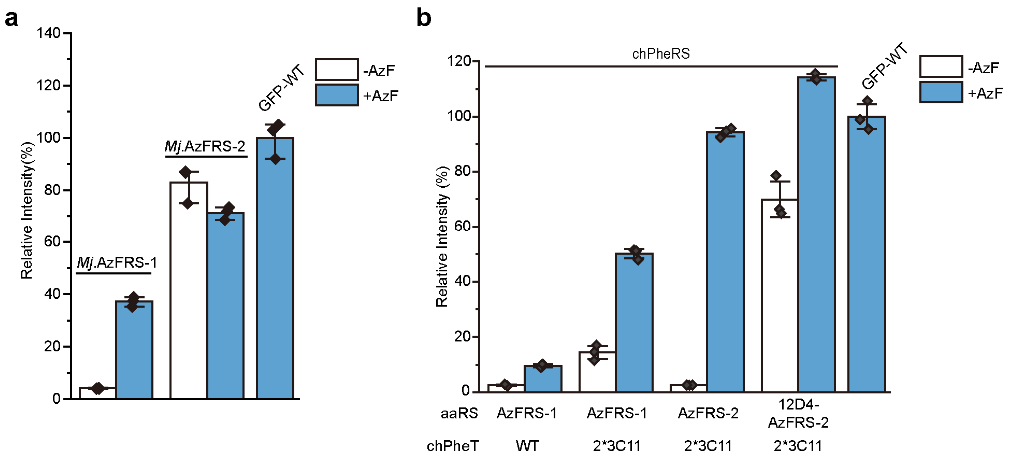

Supplementary Figure 13. The comparison of AzF incorporation systems. (a) The efficiency and background activity of the previously engineered AzF systems, *Mj*.AzFRS-1[1] and *Mj*.AzFRS-2 that is AzFRS.2.t1 in the previous paper[2]. For the protein expression, pEVOL plasmid bears *Mj*.AzFRS/tRNA pair and pBAD plasmid bears GFP reporter. (b) The efficiency and background activity of our evolved AzFRS systems, including AzFRS-1, AzFRS-2, and 12D4-AzFRS-2. For the protein expression, pBK plasmid bears AzFRS and pNEG plasmid bears tRNA and GFP reporter. Error bars represents ±standard error of the mean from three biologically independent experiments. Source data are provided as a Source Data file.

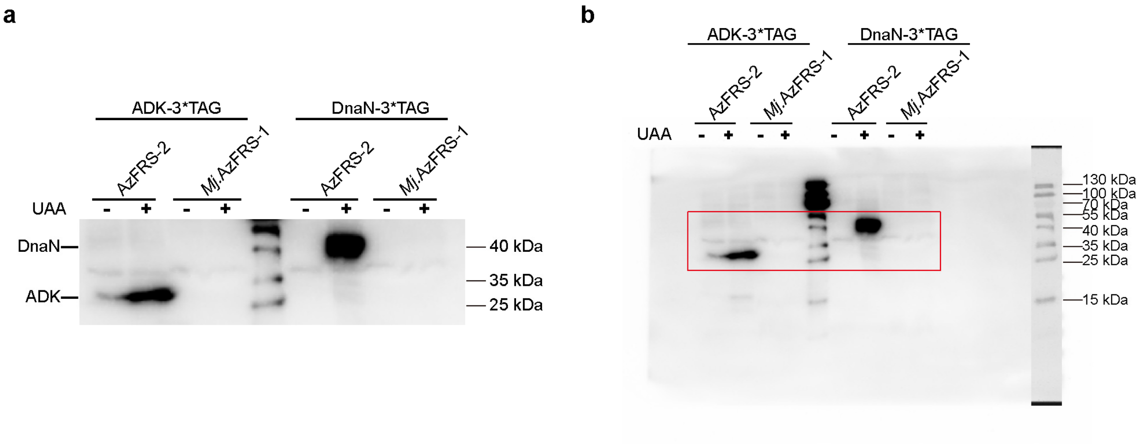

Supplementary Figure 14. Western blot analysis of three-site AzFs incorporation at essential proteins. (a) Western blot analysis of the efficiency of three-site AzFs incorporated into ADK and DnaN proteins using our AzFRS-2 system and *Mj*.AzFRS-1 system in presence and absence of AzF. (b) The full blots showed in Supplementary Figure 14a, the region in Supplementary Figure 14a was highlighted by a red solid-box. The experiment in the figure was repeated twice with similar results. Source data are provided as a Source Data file.

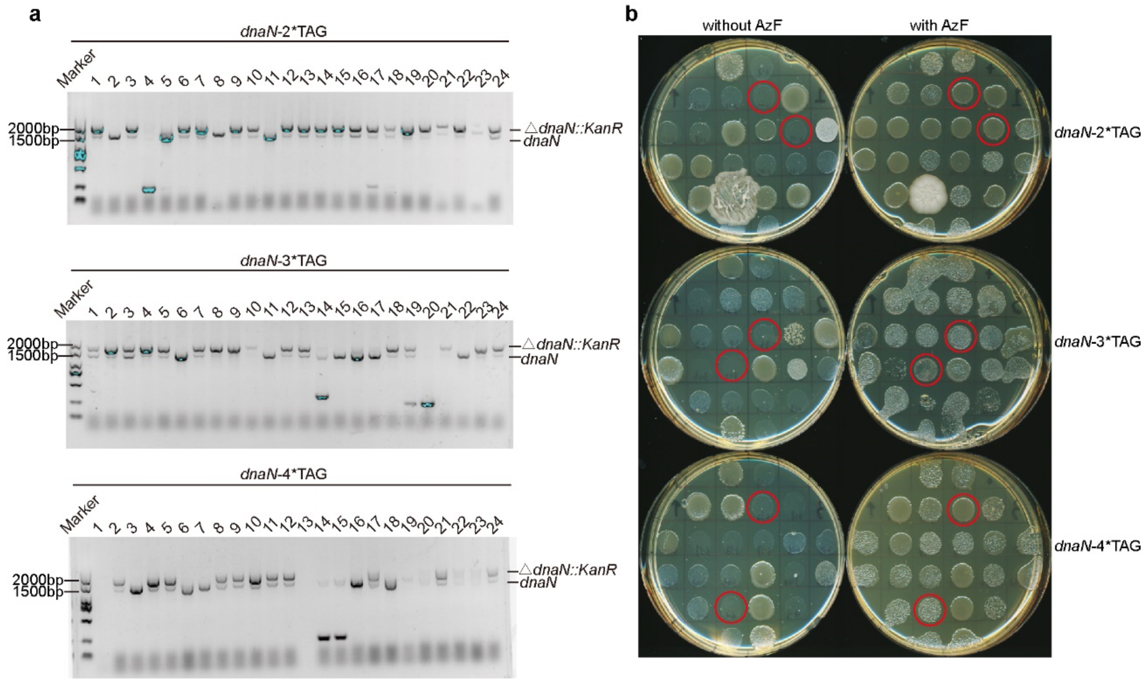

Supplementary Figure 15. Verification of knockout clones. (a) Verification of the replacement of the chromosomal *dnaN* gene by the *KanR* gene (△*dnaN*::*KanR*) via colony PCR in the indicated DH10B strains. WT-DH10B showed one major band of ~1.3 kb that corresponds to the size of the chromosomal *dnaN*, DH10B intergrated with the *KanR* fragment showed one major band of ~1.6 kb that corresponds to the △*dnaN*::*KanR*. The experiment in the figure was repeated twice with similar results. (b) Verification the viability of DH10B harboring the *dnaN*-x*TAG gene and the AzFRS-2/3C11-chPheT pair on LB-agar plates in the presence or absence of 1mM AzF. The positive strains showed robust growth in the presence of 1mM AzF while no growth was observed in the absence of AzF at 30 °C after 24 h. These positive clones are highlighted by red solid-circles.

a

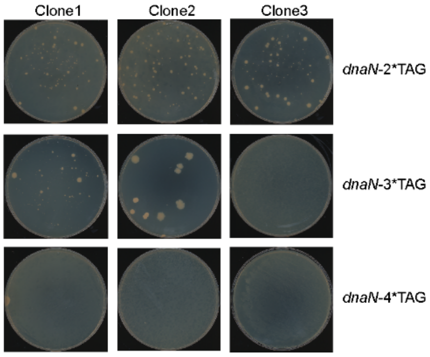

b

Strain		Total cells	Escape cells	Escape frequency
dnaN-2*TAG	Clone1	3.27E9	84	2.57E-8
	Clone2	6.20E9	86	1.39E-8
	Clone3	3.62E9	76	2.10E-8
dnaN-3*TAG	Clone1	2.80E9	48	1.71E-8
	Clone2	2.48E9	13	5.24E-9
	Clone3	3.15E9	3	0.95E-9
dnaN-4*TAG	Clone1	5.06E9	0	-
	Clone2	5.31E9	0	-
	Clone3	6.45E9	0	-

Supplementary Figure 16. Escape frequencies of auxotrophic strains. (a) Escape colonies are shown for engineered auxotrophic strains (*dnaN*-2*TAG，*dnaN*-3*TAG or *dnaN*-4*TAG) on LB-agar media in the absence of AzF. (b) Statistic table of engineered auxotrophic strains is shown with the total cells, escape cells and calculated escape frequency.

Supplementary Sequence 1. The sequences of DNA fragments used to construct UAA-dependent synthetic auxotrophs

The fragment from the upstream 100bp of essential gene is colored in blue, the downstream 100bp of essential gene is colored in orange, and the kanamycin resistance cassette is colored in black.

Upstream$_{100bp}$-Δ*dnaN::KanR*-Dowmstream$_{100bp}$

GTGCTTCATGCCTGCCGTAAGATCGAGCAGTTGCGTGAAGAGAGCCACGAT
ATCAAAGAAGATTTTTCAAATTTAATCAGAACATTGTCATCGTAAACCTGAA
GTTCCTATACTTTCTAGAGAATAGGAACTTCGGAATAGGAACTTCAAGATCC
CCTCACGCTGCCGCAAGCACTCAGGGCGCAAGGGCTGCTAAAGGAAGCGGA
ACACGTAGAAAGCCAGTCCGCAGAAACGGTGCTGACCCCGGATGAATGTCA
GCTACTGGGCTATCTGGACAAGGGAAAACGCAAGCGCAAAGAGAAAGCAG
GTAGCTTGCAGTGGGCTTACATGGCGATAGCTAGACTGGGCGGTTTTATGG
ACAGCAAGCGAACCGGAATTGCCAGCTGGGGCGCCCTCTGGTAAGGTTGGG
AAGCCCTGCAAAGTAAACTGGATGGCTTTCTTGCCGCCAAGGATCTGATGG
CGCAGGGGATCAAGATCTGATCAAGAGACAGGATGAGGATCGTTTCGCATG
ATTGAACAAGATGGATTGCACGCAGGTTCTCCGGCCGCTTGGGTGGAGAGG
CTATTCGGCTATGACTGGGCACAACAGACAATCGGCTGCTCTGATGCCGCC
GTGTTCCGGCTGTCAGCGCAGGGGCGCCCGGTTCTTTTTGTCAAGACCGACC
TGTCCGGTGCCCTGAATGAACTGCAGGACGAGGCAGCGCGGCTATCGTGGC
TGGCCACGACGGGCGTTCCTTGCGCAGCTGTGCTCGACGTTGTCACTGAAGC
GGGAAGGGACTGGCTGCTATTGGGCGAAGTGCCGGGGCAGGATCTCCTGTC
ATCTCACCTTGCTCCTGCCGAGAAAGTATCCATCATGGCTGATGCAATGCGG
CGGCTGCATACGCTTGATCCGGCTACCTGCCCATTCGACCACCAAGCGAAA
CATCGCATCGAGCGAGCACGTACTCGGATGGAAGCCGGTCTTGTCGATCAG
GATGATCTGGACGAAGAGCATCAGGGGCTCGCGCCAGCCGAACTGTTCGCC
AGGCTCAAGGCGCGCATGCCCGACGGCGAGGATCTCGTCGTGACCCATGGC

GATGCCTGCTTGCCGAATATCATGGTGGAAAATGGCCGCTTTTCTGGATTCA
TCGACTGTGGCCGGCTGGGTGTGGCGGACCGCTATCAGGACATAGCGTTGG
CTACCCGTGATATTGCTGAAGAGCTTGGCGGCGAATGGGCTGACCGCTTCCT
CGTGCTTTACGGTATCGCCGCTCCCGATTCGCAGCGCATCGCCTTCTATCGC
CTTCTTGACGAGTTCTTCTGAGCGGGACTCTGGGGTTCGAAATGACCGACCA
AGCGACGCCCAACCTGCCATCACGAGATTTCGATTCCACCGCCGCCTTCTAT
GAAAGGTTGGGCTTCGGAATCGTTTTCCGGGACGCCGGCTGGATGATCCTCC
AGCGCGGGGATCTCATGCTGGAGTTCTTCGCCCACCCCAGCTTCAAAAGCG
CTCTGAAGTTCCTATACTTTCTAGAGAATAGGAACTTCTGTCCCTCACCCGC
TTGTTGATCCGCGATTTCCGCAACATTGAAACCGCGGATCTCGCCTTATCTC
CCGGCTTTAACTTTCTGGTAGGTGCCAACGGCAG

Upstream$_{100bp}$-Δ*pgsA::KanR* -Dowmstream$_{100bp}$

CGCAAGGTCTGGCAGAAAAGATCTTCTGGTCGTTGAAACATTGATGTCTCTG
TAGCAACATAGGGGTAATCTTACTGACAACAGATAGTTACCCGTCATTGAA
GTTCCTATACTTTCTAGAGAATAGGAACTTCGGAATAGGAACTTCAAGATCC
CCTCACGCTGCCGCAAGCACTCAGGGCGCAAGGGCTGCTAAAGGAAGCGGA
ACACGTAGAAAGCCAGTCCGCAGAAACGGTGCTGACCCCGGATGAATGTCA
GCTACTGGGCTATCTGGACAAGGGAAAACGCAAGCGCAAAGAGAAAGCAG
GTAGCTTGCAGTGGGCTTACATGGCGATAGCTAGACTGGGCGGTTTTATGG
ACAGCAAGCGAACCGGAATTGCCAGCTGGGGCGCCCTCTGGTAAGGTTGGG
AAGCCCTGCAAAGTAAACTGGATGGCTTTCTTGCCGCCAAGGATCTGATGG
CGCAGGGGATCAAGATCTGATCAAGAGACAGGATGAGGATCGTTTCGCATG
ATTGAACAAGATGGATTGCACGCAGGTTCTCCGGCCGCTTGGGTGGAGAGG
CTATTCGGCTATGACTGGGCACAACAGACAATCGGCTGCTCTGATGCCGCC
GTGTTCCGGCTGTCAGCGCAGGGGCGCCCGGTTCTTTTTGTCAAGACCGACC
TGTCCGGTGCCCTGAATGAACTGCAGGACGAGGCAGCGCGGCTATCGTGGC

TGGCCACGACGGGCGTTCCTTGCGCAGCTGTGCTCGACGTTGTCACTGAAGC
GGGAAGGGACTGGCTGCTATTGGGCGAAGTGCCGGGGCAGGATCTCCTGTC
ATCTCACCTTGCTCCTGCCGAGAAAGTATCCATCATGGCTGATGCAATGCGG
CGGCTGCATACGCTTGATCCGGCTACCTGCCCATTCGACCACCAAGCGAAA
CATCGCATCGAGCGAGCACGTACTCGGATGGAAGCCGGTCTTGTCGATCAG
GATGATCTGGACGAAGAGCATCAGGGGCTCGCGCCAGCCGAACTGTTCGCC
AGGCTCAAGGCGCGCATGCCCGACGGCGAGGATCTCGTCGTGACCCATGGC
GATGCCTGCTTGCCGAATATCATGGTGGAAAATGGCCGCTTTTCTGGATTCA
TCGACTGTGGCCGGCTGGGTGTGGCGGACCGCTATCAGGACATAGCGTTGG
CTACCCGTGATATTGCTGAAGAGCTTGGCGGCGAATGGGCTGACCGCTTCCT
CGTGCTTTACGGTATCGCCGCTCCCGATTCGCAGCGCATCGCCTTCTATCGC
CTTCTTGACGAGTTCTTCTGAGCGGGACTCTGGGGTTCGAAATGACCGACCA
AGCGACGCCCAACCTGCCATCACGAGATTTCGATTCCACCGCCGCCTTCTAT
GAAAGGTTGGGCTTCGGAATCGTTTTCCGGGACGCCGGCTGGATGATCCTCC
AGCGCGGGGATCTCATGCTGGAGTTCTTCGCCCACCCCAGCTTCAAAAGCG
CTCTGAAGTTCCTATACTTTCTAGAGAATAGGAACTTCTCGTTTCGGCGTAA
TTTTCAGCAAACGATCAAAAGTGGTGAAAAATATCGTTGACTCATCGCGCC
AGGTAAGTAGAATGCAACGCATCGAACGGCGGCAC

Upstream$_{100bp}$-△*adk::KanR* -Dowmstream$_{100bp}$

CCGTTTCAGCCCCAGGTGCCTTTCTTGAGGCAATCGCCTGTTGGTGGTATCG
TTTATCGCTTTTTCAAAAAATTCGACACATTTTAAGGGGATTTTCGCAGAAG
TTCCTATACTTTCTAGAGAATAGGAACTTCGGAATAGGAACTTCAAGATCCC
CTCACGCTGCCGCAAGCACTCAGGGCGCAAGGGCTGCTAAAGGAAGCGGA
ACACGTAGAAAGCCAGTCCGCAGAAACGGTGCTGACCCCGGATGAATGTCA
GCTACTGGGCTATCTGGACAAGGGAAAACGCAAGCGCAAAGAGAAAGCAG
GTAGCTTGCAGTGGGCTTACATGGCGATAGCTAGACTGGGCGGTTTTATGG

ACAGCAAGCGAACCGGAATTGCCAGCTGGGGCGCCCTCTGGTAAGGTTGGG
AAGCCCTGCAAAGTAAACTGGATGGCTTTCTTGCCGCCAAGGATCTGATGG
CGCAGGGGATCAAGATCTGATCAAGAGACAGGATGAGGATCGTTTCGCATG
ATTGAACAAGATGGATTGCACGCAGGTTCTCCGGCCGCTTGGGTGGAGAGG
CTATTCGGCTATGACTGGGCACAACAGACAATCGGCTGCTCTGATGCCGCC
GTGTTCCGGCTGTCAGCGCAGGGGCGCCCGGTTCTTTTTGTCAAGACCGACC
TGTCCGGTGCCCTGAATGAACTGCAGGACGAGGCAGCGCGGCTATCGTGGC
TGGCCACGACGGGCGTTCCTTGCGCAGCTGTGCTCGACGTTGTCACTGAAGC
GGGAAGGGACTGGCTGCTATTGGGCGAAGTGCCGGGGCAGGATCTCCTGTC
ATCTCACCTTGCTCCTGCCGAGAAAGTATCCATCATGGCTGATGCAATGCGG
CGGCTGCATACGCTTGATCCGGCTACCTGCCCATTCGACCACCAAGCGAAA
CATCGCATCGAGCGAGCACGTACTCGGATGGAAGCCGGTCTTGTCGATCAG
GATGATCTGGACGAAGAGCATCAGGGGCTCGCGCCAGCCGAACTGTTCGCC
AGGCTCAAGGCGCGCATGCCCGACGGCGAGGATCTCGTCGTGACCCATGGC
GATGCCTGCTTGCCGAATATCATGGTGGAAAATGGCCGCTTTTCTGGATTCA
TCGACTGTGGCCGGCTGGGTGTGGCGGACCGCTATCAGGACATAGCGTTGG
CTACCCGTGATATTGCTGAAGAGCTTGGCGGCGAATGGGCTGACCGCTTCCT
CGTGCTTTACGGTATCGCCGCTCCCGATTCGCAGCGCATCGCCTTCTATCGC
CTTCTTGACGAGTTCTTCTGAGCGGGACTCTGGGGTTCGAAATGACCGACCA
AGCGACGCCCAACCTGCCATCACGAGATTTCGATTCCACCGCCGCCTTCTAT
GAAAGGTTGGGCTTCGGAATCGTTTTCCGGGACGCCGGCTGGATGATCCTCC
AGCGCGGGGATCTCATGCTGGAGTTCTTCGCCCACCCCAGCTTCAAAAGCG
CTCTGAAGTTCCTATACTTTCTAGAGAATAGGAACTTCTTCGAAAGCGCGCA
CGGACAGTCCCCTCGCCCCCTCGGGGAGAGGGTTAGGGTGAGGGGAACAGG
CCCGCACAAGCAAACTTATCAGCAATCTCAGGCCG

Upstream$_{100bp}$-△*TyrS::KanR*-Dowmstream$_{100bp}$

TGGTACTCCTGATTCTGGCACTTTATTCTATGTCTCTTTCGCATCTGGCGAAA
AGTCGTGTACCGGCAAAGGTGCAGTCGTTATATACATGGAGATTTTGGAAG
TTCCTATACTTTCTAGAGAATAGGAACTTCGGAATAGGAACTTCAAGATCCC
CTCACGCTGCCGCAAGCACTCAGGGCGCAAGGGCTGCTAAAGGAAGCGGA
ACACGTAGAAAGCCAGTCCGCAGAAACGGTGCTGACCCCGGATGAATGTCA
GCTACTGGGCTATCTGGACAAGGGAAAACGCAAGCGCAAAGAGAAAGCAG
GTAGCTTGCAGTGGGCTTACATGGCGATAGCTAGACTGGGCGGTTTTATGG
ACAGCAAGCGAACCGGAATTGCCAGCTGGGGCGCCCTCTGGTAAGGTTGGG
AAGCCCTGCAAAGTAAACTGGATGGCTTTCTTGCCGCCAAGGATCTGATGG
CGCAGGGGATCAAGATCTGATCAAGAGACAGGATGAGGATCGTTTCGCATG
ATTGAACAAGATGGATTGCACGCAGGTTCTCCGGCCGCTTGGGTGGAGAGG
CTATTCGGCTATGACTGGGCACAACAGACAATCGGCTGCTCTGATGCCGCC
GTGTTCCGGCTGTCAGCGCAGGGGCGCCCGGTTCTTTTTGTCAAGACCGACC
TGTCCGGTGCCCTGAATGAACTGCAGGACGAGGCAGCGCGGCTATCGTGGC
TGGCCACGACGGGCGTTCCTTGCGCAGCTGTGCTCGACGTTGTCACTGAAGC
GGGAAGGGACTGGCTGCTATTGGGCGAAGTGCCGGGGCAGGATCTCCTGTC
ATCTCACCTTGCTCCTGCCGAGAAAGTATCCATCATGGCTGATGCAATGCGG
CGGCTGCATACGCTTGATCCGGCTACCTGCCCATTCGACCACCAAGCGAAA
CATCGCATCGAGCGAGCACGTACTCGGATGGAAGCCGGTCTTGTCGATCAG
GATGATCTGGACGAAGAGCATCAGGGGCTCGCGCCAGCCGAACTGTTCGCC
AGGCTCAAGGCGCGCATGCCCGACGGCGAGGATCTCGTCGTGACCCATGGC
GATGCCTGCTTGCCGAATATCATGGTGGAAAATGGCCGCTTTTCTGGATTCA
TCGACTGTGGCCGGCTGGGTGTGGCGGACCGCTATCAGGACATAGCGTTGG
CTACCCGTGATATTGCTGAAGAGCTTGGCGGCGAATGGGCTGACCGCTTCCT
CGTGCTTTACGGTATCGCCGCTCCCGATTCGCAGCGCATCGCCTTCTATCGC
CTTCTTGACGAGTTCTTCTGAGCGGGACTCTGGGGTTCGAAATGACCGACCA
AGCGACGCCCAACCTGCCATCACGAGATTTCGATTCCACCGCCGCCTTCTAT

GAAAGGTTGGGCTTCGGAATCGTTTTCCGGGACGCCGGCTGGATGATCCTCC
AGCGCGGGGATCTCATGCTGGAGTTCTTCGCCCACCCCAGCTTCAAAAGCG
CTCTGAAGTTCCTATACTTTCTAGAGAATAGGAACTTCTGCATTAAGTGGAA
AGGGGGAGTGAGAAATCACTCCCCCTGGTTTTATACAGGGAACATGATGA
AAAATATTCTCGCTATCCAGTCTCACGTTGTTTAT

Supplementary Sequence 2. The DNA sequences of OTS integration cassette

The DNA sequence of OTS integration cassette 1

Homology arms to the genomic integration site 1 (at position 17231-17232 in DH10B) are highlighted in yellow. ChPheT is underlined with dot line. 12D4-AzFRS-2 is underlined with wavy line. The chloramphenicol resistance cassette is colored in green.

AGTAAGAGGCACTCTACATGTGTTCAGCATATAGGAGGCCTCGGGTTGATG
GTAAAATATCACTCGGGGCTTTTCTCTATCTGCCGTTCAGCTAATGCCTGAG
ACAGACAGCCTCAAGCACCCGCCGCTATTATATCGCTCTCTTTAACCCATTT
TGTTTTATCGATTCTAATCCTGAAGACGCCTCGCATTTTTGTGGCGTAATTTT
TTAATGATTTAATTATTTAACTTTAATTTATCTCTTCATCGCAATTATTGACG
ACAAGCTGGATTATTTTTGAAATATTGGCCTAACAAGCATTTTCCCAGTCAC
GACGTTGTAAAACGACGGCCAGTGCCAAGCTTAAAAAAAATCCTTAGCTTT
CGCTAAGGATCTGCAGTGGTGTGAGAGCCGGGAATCTAACCCGGCTGAACG
GATTTAGAGTCCGTTCGATCTACATGATCACTCTCACGAATTCAGCGTTACA
AGTATTACACAAAGTTTTTATGTTGAGAATATTTTTTTGATGGGACTAGTTA
AAAAAAATCCTTAGCTTTCGCTAAGGATCTGCAGTGGTGTGAGAGCCGGGA
ATCTAACCCGGCTGAACGGATTTAGAGTCCGTTCGATCTACATGATCACTCT
CACGAATTCAGCGTTACAAGTATTACACAAAGTTTTTATGTTGAGAATATT
TTTTTGATGGGGCGCCACTTATTTTTGATCGTTCGCTCAAAGAAGCGGCGCC
AGGGTTGTTTTTCTTTTCACCAGTGAGACGGGCAACAGAACGCCATGAGCG
GCCTCATTTCTTATTCTGAGTTACAACAGTCCGCACCGCTGCCGGTAGCTCC

TTCCGGTGGGCGCGGGGCATGACTATCGTCGCCGCACTTATGACTGTCTTCT
TTATCATGCAACTCGTAGGACAGGTGCCGGCAGCGCCCAACAGTCCCCCGG
CCACGGGGCCTGCCACCATACCCACGCCGAAACAAGCGCCCTGCACCATTA
TGTTCCGGATCTGCATCGCAGGATGCTGCTGGCTACCCTGTGGAACACCTAC
ATCTGTATTAACGAAGCGCTAACCGTTTTTATCATGCTCTGGGAGGCAGAAT
AAATGGCTGAGTTGAAGGATCCTCGGGTTGTCAGCCTGTCCCGCTTATAAAA
GCTGTTGTGACCGCTTGCTCTAGCCAGCTATCGAGTTGTGAACCGATCCATC
TAGCAATTGGTCTCGATCTAGCGATAGGCTTCGATCTAGCTATGTAGAAACG
CCGTGTGCTCGATCGCTTGATAAGGTCCACGTAGCTGCTATAATTGCTTCAA
CAGAACATATTGACTATCCGGTATTACCCGGCCGCCGTTATACGTTGTTTAC
GCTTTGAGGAATCCCATATGGATAAGAAGCCGCTGGATGTTCTGATCTCTGC
GACCGGTCTGTGGATGTCCCGTACCGGCACGCTGCACAAGATCAAGCACTA
TGAGATTTCTCGTTCTAAAATCTACATCGAAATGGCGTGTGGTGACCATCTG
GTTGTGAACAACTCTCGTTCTTGTCGTCCCGCACGTGCATTCCGTTATCATA
AATACCGTAAAACCTGCAAACGTTGTCGTGTTTCTGACGAAGATATCAACA
ACTTCCTGACCCGTTCTACCGAAGGCAAAACCTCTGTTAAAGTTAAAGTTGT
TTCTGAGCCGAAAGTGAAAAAAGCGATGCCGAAATCTGTTTCTCGTGCGCC
GAAACCGCTGGAAAATCCGGTTTCTGCGAAAGCGTCTACCGACACCTCTCG
TTCTGTTCCGTCTCCGGCGAAATCTACCCCGAACTCTCCGGTTCCGACCTCT
GCAAGCGCCCCAGCTCTGACTAAATCCCAGACGGACCGTCTGGAGGTGCTG
CTGAACCCAAAGGATGAAATCTCTCTGAACAGCGGCAAGCCTTTCCGTGAG
CTGGAAAGCGAGCTGCTGTCTCGTCGTAAAAAGGATCTGCAACAGATCTAC
GCTGAGGAACGCGAGGGTGGCGGAAGCGGCGGCGGAAGCCAGGCCTGGGG
ATCGAGGCCTCCTGCAGCAGAGTGTGCCACCCAAAGAGCTCCAGGCAGTGT
GGTGGAGCTGCTGGGCAAATCCTACCCTCAGGACGACCACAGCAACCTCAC
CCGGAAGGTCCTCACCAGAGTTGGCAGGAACCTGCACAACCAGCAGCATCA
CCCTCTGTGGCTGATCAAGGAGAGGGTGTTGGAGCACTTCAACAAGCAGTA

TGTGGGCAGCTCTGGGACCCCGTTGTTCTCGGTCTATGACAACCTTTCGCCA
GTGGTCACGACCTGGCAGAACTTTGACAGCCTGCTCATCCCAGCTGATCACC
CCTGCAGGAAGAAGGGGGACAACTATTACCTGAATCGGACTCACATGCTGA
GATCCCACACGTCCGCACACCAGTGGGACTTGCTGCACGCGGGACTGGATG
CCTTCCTGGTGGTGGGTGATGTCTACAGGCGTGACCAGATCGACTCCCAGCA
CTACCCTATTTTCCACCAGCTGGAGGCCGTGCGGCTCTTCACCAAGCATGAG
TTATTTGCTGGTATAAAGGATGGGGAAAGCCTGCAGCTCTTTGAACAAAGTT
CTCGCTCTGCGCATAAACAAGAGACACACACCATGGAGGCCGTGAAGCTTG
TTGAGTTTGATCTTAAGCAAACGCTTACCAGGCTCATGGCACATCTTTTTGG
AGATGAGCCGGAGATAAGGTGGGTAGACTGCTACATTCCTTTTGGACATCC
TTCCTTTGAGATGGAGATCAACTTTCATGGAGAATGGCTGGAAGTTCTTGGC
TGCGGGGTGTTGGAACAACAACTGGTCAATTCAGCTGGTGCTCAAGACCGA
ATCGGCTGGGGATTTGGCCTAGGGTTAGAAAGGCTAGCCATGATCCTCTAC
GACATCCCTGATATCCGTCTCTTCTGGTGTGAGGACGAGCGCTTCCTGAAGC
AGTTCTGTGTATCCAACATTAATCAGAAGGTGAAGTTTCAGCCTCTTAGCAA
ATAACTGCAGTTTCAAACGCTAAATTGCCTGATGCGCTACGCTTATCAGGCC
TACATGATCTCTGCAATATATTGAGTTTGCGTGCTTTTGTAGGCCGGATAAG
GCGTTCACGCCGCATCCGGCAAGAAACAGCAAACAATCCAAAACGCCGCGT
TCAGCGGCGTTTTTCTGCTTTTCTTCGCGAATTAATTCCGCTTCGCACATGT
GAGCAAAAGGCCAGCAAAAGGCCAGATCATATCGTCAATTATTACCTCCAC
GGGGAGAGCCTGAGCAAACTGGCCTCAGGCATTTGAGAAGCACACGGTCAC
ACTGCTTCCGGTAGTCAATAAACCGGTAAACCAGCAATAGACATAAGCGGC
TATTTAACGACCCTGCCCTGAACCGACGACCGGGTCGAATTTGCTTTCGAAT
TTCTGCCATTCATCCGCTTATTATCACTTATTCAGGCGTAGCAACCAGGCGT
TTAAGGGCACCAATAACTGCCTTAAAAAAATTACGCCCCGCCCTGCCACTC
ATCGCAGTACTGTTGTAATTCATTAAGCATTCTGCCGACATGGAAGCCATCA
CAAACGGCATGATGAACCTGAATCGCGAATTTGCTTTCGAATTTCTGCCATT

CATCCGCTTATTATCACTTATTCAGGCGTAGCAACCAGGCGTTTAAGGGCAC
CAATAACTGCCTTAAAAAAATTACGCCCCGCCCTGCCACTCATCGCAGTACT
GTTGTAATTCATTAAGCATTCTGCCGACATGGAAGCCATCACAAACGGCAT
GATGAACCTGAATCGCCAGCGGCATCAGCACCTTGTCGCCTTGCGTATAATA
TTTGCCCATGGTGAAAACGGGGGCGAAGAAGTTGTCCATATTGGCCACGTT
TAAATCAAAACTGGTGAAACTCACCCAGGGATTGGCTGAGACGAAAAACAT
ATTCTCAATAAACCCTTTAGGGAAATAGGCCAGGTTTTCACCGTAACACGCC
ACATCTTGCGAATATATGTGTAGAAACTGCCGGAAATCGTCGTGGTATTCAC
TCCAGAGCGATGAAAACGTTTCAGTTTGCTCATGGAAAACGGTGTAACAAG
GGTGAACACTATCCCATATCACCAGCTCACCGTCTTTCATTGCCATACGGAA
TTCCGGATGAGCATTCATCAGGCGGGCAAGAATGTGAATAAAGGCCGGATA
AAACTTGTGCTTATTTTTCTTTACGGTCTTTAAAAAGGCCGTAATATCCAGCT
GAACGGTCTGGTTATAGGTACATTGAGCAACTGACTGAAATGCCTCAAAAT
GTTCTTTACGATGCCATTGGGATATATCAACGGTGGTATATCCAGTGATTTT
TTTCTCCATTTTAGCTTCCTTAGCTCCTGAAAATCTCGATAACTCAAAAAAT
ACGCCCGGTAGTGATCTTATTTCATTATGGTGAAAGTTGGAACCTCTTACGT
GCCGATCAACGTCTCATTTTCGCCAAAAGTTGGCCCAGGGCTTCCCGGTATC
AACAGGGACACCAGGATTTATTTATTCTGCGAAGTCGCCGACTGACAACAA
ATTAATTATTACTTTTCCTAATTAATCCCTCAGGAATCCTCACCTTAAGCTAT
GATTATCTAGGCTTAGGGTCACTCGTGAGCGCTTACAGCCGTCAAAAACGC
ATCTCACCGCTGATGGCGCAAATTCTTCAATAGCTCGTAAAAAACGAATTAT
TCCTACACTATAATCTGATTTTAACGATGATTCGTGCGGGGTAAAATAGTAA
AAACGATCTATTCACCTGAAAGAGAAATAAAAAGTGAAACATCTGCATCGA
TTCTTTAGCAGTGATGCCTCGGGA

The DNA sequence of OTS integration cassette 2

Homology arms to the genomic integration site 2 (at position 2048790-2048791 in DH10B) are highlighted in yellow. ChPheT is underlined with dot line. 12D4-AzFRS-2 is underlined with wavy line. The kanamycin resistance cassette is colored in blue.

AACAACTTTGCAGATTAATTAACCAATTGAAATGACTTATGAAATTTAGTGT
TGACAGACAAGGTACCGCTAAGTAATATTCGCCCCGTTCACACGATTCCTCT
GTAGTTCAGTCGGTAGAACGGCGGACTGTTAATCCGTATGTCACTGGTTCGA
GTCCAGTCAGAGGAGCCAAATTCTAAAAATTCGCTTTTTAGCGCAATGTCA
CTGACCTTAGTTGAACATTGTTTTTAACGGATAGCGGGTTTTAACATCTTA
AGCGCCCTCGACCTTTATGGTTGAGGGCGTTTTGCTATGTTTTCCCAGTCAC
GACGTTGTAAAACGACGGCCAGTGCCAAGCTTAAAAAAATCCTTAGCTTT
CGCTAAGGATCTGCAGTGGTGTGAGAGCCGGGAATCTAACCCGGCTGAACG
GATTTAGAGTCCGTTCGATCTACATGATCACTCTCACGAATTCAGCGTTACA
AGTATTACACAAAGTTTTTATGTTGAGAATATTTTTTTGATGGGACTAGTTA
AAAAAAATCCTTAGCTTTCGCTAAGGATCTGCAGTGGTGTGAGAGCCGGGA
ATCTAACCCGGCTGAACGGATTTAGAGTCCGTTCGATCTACATGATCACTCT
CACGAATTCAGCGTTACAAGTATTACACAAAGTTTTTATGTTGAGAATATT
TTTTTGATGGGGCGCCACTTATTTTTGATCGTTCGCTCAAAGAAGCGGCGCC
AGGGTTGTTTTTCTTTTCACCAGTGAGACGGGCAACAGAACGCCATGAGCG
GCCTCATTTCTTATTCTGAGTTACAACAGTCCGCACCGCTGCCGGTAGCTCC
TTCCGGTGGGCGCGGGGCATGACTATCGTCGCCGCACTTATGACTGTCTTCT
TTATCATGCAACTCGTAGGACAGGTGCCGGCAGCGCCCAACAGTCCCCCGG
CCACGGGGCCTGCCACCATACCCACGCCGAAACAAGCGCCCTGCACCATTA
TGTTCCGGATCTGCATCGCAGGATGCTGCTGGCTACCCTGTGGAACACCTAC
ATCTGTATTAACGAAGCGCTAACCGTTTTTATCATGCTCTGGGAGGCAGAAT
AAATGGCTGAGTTGAAGGATCCTCGGGTTGTCAGCCTGTCCCGCTTATAAAA
GCTGTTGTGACCGCTTGCTCTAGCCAGCTATCGAGTTGTGAACCGATCCATC
TAGCAATTGGTCTCGATCTAGCGATAGGCTTCGATCTAGCTATGTAGAAACG

CCGTGTGCTCGATCGCTTGATAAGGTCCACGTAGCTGCTATAATTGCTTCAA
CAGAACATATTGACTATCCGGTATTACCCGGCCGCCGTTATACGTTGTTTAC
GCTTTGAGGAATCCCATATGGATAAGAAGCCGCTGGATGTTCTGATCTCTGC
GACCGGTCTGTGGATGTCCCGTACCGGCACGCTGCACAAGATCAAGCACTA
TGAGATTTCTCGTTCTAAAATCTACATCGAAATGGCGTGTGGTGACCATCTG
GTTGTGAACAACTCTCGTTCTTGTCGTCCCGCACGTGCATTCCGTTATCATA
AATACCGTAAAACCTGCAAACGTTGTCGTGTTTCTGACGAAGATATCAACA
ACTTCCTGACCCGTTCTACCGAAGGCAAAACCTCTGTTAAAGTTAAAGTTGT
TTCTGAGCCGAAAGTGAAAAAAGCGATGCCGAAATCTGTTTCTCGTGCGCC
GAAACCGCTGGAAAATCCGGTTTCTGCGAAAGCGTCTACCGACACCTCTCG
TTCTGTTCCGTCTCCGGCGAAATCTACCCCGAACTCTCCGGTTCCGACCTCT
GCAAGCGCCCCAGCTCTGACTAAATCCCAGACGGACCGTCTGGAGGTGCTG
CTGAACCCAAAGGATGAAATCTCTCTGAACAGCGGCAAGCCTTTCCGTGAG
CTGGAAAGCGAGCTGCTGTCTCGTCGTAAAAAGGATCTGCAACAGATCTAC
GCTGAGGAACGCGAGGGTGGCGGAAGCGGCGGCGGAAGCCAGGCCTGGGG
ATCGAGGCCTCCTGCAGCAGAGTGTGCCACCCAAAGAGCTCCAGGCAGTGT
GGTGGAGCTGCTGGGCAAATCCTACCCTCAGGACGACCACAGCAACCTCAC
CCGGAAGGTCCTCACCAGAGTTGGCAGGAACCTGCACAACCAGCAGCATCA
CCCTCTGTGGCTGATCAAGGAGAGGGTGTTGGAGCACTTCAACAAGCAGTA
TGTGGGCAGCTCTGGGACCCCGTTGTTCTCGGTCTATGACAACCTTTCGCCA
GTGGTCACGACCTGGCAGAACTTTGACAGCCTGCTCATCCCAGCTGATCACC
CCTGCAGGAAGAAGGGGGACAACTATTACCTGAATCGGACTCACATGCTGA
GATCCCACACGTCCGCACACCAGTGGGACTTGCTGCACGCGGGACTGGATG
CCTTCCTGGTGGTGGGTGATGTCTACAGGCGTGACCAGATCGACTCCCAGCA
CTACCCTATTTTCCACCAGCTGGAGGCCGTGCGGCTCTTCACCAAGCATGAG
TTATTTGCTGGTATAAAGGATGGGGAAAGCCTGCAGCTCTTTGAACAAAGTT
CTCGCTCTGCGCATAAACAAGAGACACACACCATGGAGGCCGTGAAGCTTG

TTGAGTTTGATCTTAAGCAAACGCTTACCAGGCTCATGGCACATCTTTTTGG
AGATGAGCCGGAGATAAGGTGGGTAGACTGCTACATTCCTTTTGGACATCC
TTCCTTTGAGATGGAGATCAACTTTCATGGAGAATGGCTGGAAGTTCTTGGC
TGCGGGGTGTTGGAACAACAACTGGTCAATTCAGCTGGTGCTCAAGACCGA
ATCGGCTGGGGATTTGGCCTAGGGTTAGAAAGGCTAGCCATGATCCTCTAC
GACATCCCTGATATCCGTCTCTTCTGGTGTGAGGACGAGCGCTTCCTGAAGC
AGTTCTGTGTATCCAACATTAATCAGAAGGTGAAGTTTCAGCCTCTTAGCAA
ATAACTGCAGTTTCAAACGCTAAATTGCCTGATGCGCTACGCTTATCAGGCC
TACATGATCTCTGCAATATATTGAGTTTGCGTGCTTTTGTAGGCCGGATAAG
GCGTTCACGCCGCATCCGGCAAGAAACAGCAAACAATCCAAAACGCCGCGT
TCAGCGGCGTTTTTTCTGCTTTTCTTCGCGAATTAATTCCGCTTCGCACATGT
GAGCAAAAGGCCAGCAAAAGGCCAGATCATATCGTCAATTATTACCTCCAC
GGGGAGAGCCTGAGCAAACTGGCCTCAGGCATTTGAGAAGCACACGGTCAC
ACTGCTTCCGGTAGTCAATAAACCGGTAAACCAGCAATAGACATAAGCGGC
TATTTAACGACCCTGCCCTGAACCGACGACCGGGTCGAATTTGCTTTCGAAT
TTCTGCCATTCATCCGCTTATTATCACTTATTCAGGCGTAGCAACCAGGCGT
TTAAGGGCACCAATAACTGCCTTAAAAAAATTACGCCCCGCCCTGCCACTC
ATCGCAGTACTGTTGTAATTCATTAAGCATTCTGCCGACATGGAAGCCATCA
CAAACGGCATGATGAACCTGAATCGCGAATTTGCTTTCGAATTTCTGCCATT
CATCCGCTTATTATCACTTATTCAGGCGTAGCAACCAGGCGTTTAAGGGCAC
CAATAACTGCCTTAAAAAAATCAGAAGAACTCGTCAAGAAGGCGATAGAA
GGCGATGCGCTGCGAATCGGGAGCGGCGATACCGTAAAGCACGAGGAAGC
GGTCAGCCCATTCGCCGCCAAGCTCTTCAGCAATATCACGGGTAGCCAACG
CTATGTCCTGATAGCGGTCCGCCACACCCAGCCGGCCACAGTCGATGAATC
CAGAAAAGCGGCCATTTTCCACCATGATATTCGGCAAGCAGGCATCGCCAT
GGGTCACGACGAGATCCTCGCCGTCGGGCATGCGCGCCTTGAGCCTGGCGA
ACAGTTCGGCTGGCGCGAGCCCCTGATGCTCTTCGTCCAGATCATCCTGATC

GACAAGACCGGCTTCCATCCGAGTACGTGCTCGCTCGATGCGATGTTTCGCT
TGGTGGTCGAATGGGCAGGTAGCCGGATCAAGCGTATGCAGCCGCCGCATT
GCATCAGCCATGATGGATACTTTCTCGGCAGGAGCAAGGTGAGATGACAGG
AGATCCTGCCCCGGCACTTCGCCCAATAGCAGCCAGTCCCTTCCCGCTTCAG
TGACAACGTCGAGCACAGCTGCGCAAGGAACGCCCGTCGTGGCCAGCCACG
ATAGCCGCGCTGCCTCGTCCTGCAGTTCATTCAGGGCACCGGACAGGTCGGT
CTTGACAAAAAGAACCGGGCGCCCCTGCGCTGACAGCCGGAACACGGCGGC
ATCAGAGCAGCCGATTGTCTGTTGTGCCCAGTCATAGCCGAATAGCCTCTCC
ACCCAAGCGGCCGGAGAACCTGCGTGCAATCCATCTTGTTCAATCATGCGA
AACGATCCTCATCCTGTCTCTTGATCAGATCTTGATCCCCTGCGCCATCAGA
TCCTTGGCGGCAAGAAAGCCATCCAGTTTACTTTGCAGGGCTTCCCAACCTT
ACCAGAGGGCGCCCCAGCTGGCAATTCCGGACGTCTCATTTTCGCCAAAAG
TTGGCCCAGGGCTTCCCGGTATCAACAGGGACACCAGGATTTATTTATTCTG
CGAAGAACGCCATCACCATTTTCCCCTCGATTATAAAACTTGAGTTATTCAG
TAGTCTCCCCTCTTGCAACTCACACCCAAAACTGCCTAACGAAAAGTTATTA
ATTTTCAATCATATTGCTATCAGTATTTACATTTTTTCGCTGTGCTAGAAAGG
GCGCATTTATGTTAGCTCGTTCAGGGAAGGTAAGCATGGCTACGAAGAAGA
GAAGTGGAGAAGAAATAAATGACCGACAAATATTATGCGGGATGGGAATT
AAACTACGCCGCTTAACTGCGGGTATCTGTCTGATAACTCAACTTGC

Supplementary Sequence 3. The sequences of promoter in this study

Oxb20

AAGCTGTTGTGACCGCTTGCTCTAGCCAGCTATCGAGTTGTGAACCGATCCA
TCTAGCAATTGGTCTCGATCTAGCGATAGGCTTCGATCTAGCTATGTAGAAA
CGCCGTGTGCTCGATCGCTTGATAAGGTCCACGTAGCTGCTATAATTGCTTC
AACAGAACATATTGACTATCCGGTATTACCCGGC

Oxb15

CAGACCTTGTGGCAACAATTTCTACAAAACACTTGATACTGTAAAAAAAAA

CAGTATAATTGCTTCAACAGAACATATTGACTATCCGGTATTACCCGGCATG

ACAGGAGTAAAAATGGCTATC

Glns

TTGTCAGCCTGTCCCGCTTTAATATC

Supplementary Sequence 4. The DNA sequences of chPheTs obtained from selection

chPheT-1C6

GTGAATGTGATCATGTAGATCGAACGGACTCTAAATCCGTTCAGCCGGGTT

AGATTCCCGGCGTTCACACCA

chPheT-2F6

GTGAAAGTGATCATGTAGATCGAACGGACTCTAAATCCGTTCAGCCGGGTT

AGATTCCCGGCTTTCACACCA

chPheT-3C11

GTGAGAGTGATCATGTAGATCGAACGGACTCTAAATCCGTTCAGCCGGGTT

AGATTCCCGGCTCTCACACCA

chPheT-4E3

GTATCTGTGATCATGTAGATCGAACGGACTCTAAATCCGTTCAGCCGGGTTA

GATTCCCGGCAGATACACCA

chPheT-11H1

GTAAAAGTGATCATGTAGATCGAACGGACTCTAAATCCGTTCAGCCGGGTT

AGATTCCCGGCTTTTACACCA

chPheT-16C2

GTGTTTGTGATCATGTAGATCGAACGGACTCTAAATCCGTTCAGCCGGGTTA

GATTCCCGGCAAACACACCA

Supplementary Sequence 5. The DNA and protein sequences of the evolved chPheRS

The fragment from PylRS is colored in red, the catalytic domain of canonical synthetase is colored in blue, and the linker is colored in black. Mutation sites for activity improvement are highlighted in yellow. Mutation sites for incorporation of non-natural amino acids are highlighted in magenta.

12D4-AzFRS-1 DNA sequence

ATGGATAAGAAGCCGCTGGATGTTCTGATCTCTGCGACCGGTCTGTGGATGT
CCCGTACCGGCACGCTGCACAAGATCAAGCACTATGAGATTTCTCGTTCTAA
AATCTACATCGAAATGGCGTGTGGTGACCATCTGGTTGTGAACAACTCTCGT
TCTTGTCGTCCCGCACGTGCATTCCGTTATCATAAATACCGTAAAACCTGCA
AACGTTGTCGTGTTTCTGACGAAGATATCAACAACTTCCTGACCCGTTCTAC
CGAAGGCAAAACCTCTGTTAAAGTTAAAGTTGTTTCTGAGCCGAAAGTGAA
AAAAGCGATGCCGAAATCTGTTTCTCGTGCGCCGAAACCGCTGGAAAATCC
GGTTTCTGCGAAAGCGTCTACCGACACCTCTCGTTCTGTTCCGTCTCCGGCG
AAATCTACCCCGAACTCTCCGGTTCCGACCTCTGCAAGCGCCCCAGCTCTGA
CTAAATCCCAGACGGACCGTCTGGAGGTGCTGCTGAACCCAAAGGATGAAA
TCTCTCTGAACAGCGGCAAGCCTTTCCGTGAGCTGGAAAGCGAGCTGCTGTC
TCGTCGTAAAAAGGATCTGCAACAGATCTACGCTGAGGAACGCGAGGGTGG
CGGAAGCGGCGGCGGAAGCCAGGCCTGGGGATCGAGGCCTCCTGCAGCAG
AGTGTGCCACCCAAAGAGCTCCAGGCAGTGTGGTGGAGCTGCTGGGCAAAT
CCTACCCTCAGGACGACCACAGCAACCTCACCCGGAAGGTCCTCACCAGAG
TTGGCAGGAACCTGCACAACCAGCAGCATCACCCTCTGTGGCTGATCAAGG
AGAGGGTGTTGGAGCACTTCAACAAGCAGTATGTGGGCAGCTCTGGGACCC
CGTTGTTCTCGGTCTATGACAACCTTTCGCCAGTGGTCACGACCTGGCAGAA
CTTTGACAGCCTGCTCATCCCAGCTGATCACCCCTGCAGGAAGAAGGGGGA
CAACTATTACCTGAATCGGACTCACATGCTGAGAGCGCACACGTCCGCACA
CCAGTGGGACTTGCTGCACGCGGGACTGGATGCCTTCCTGGTGGTGGGTGA

TGTCTACAGGCGTGACCAGATCGACTCCCAGCACTACCCTATTTTCCACCAG
CTGGAGGCCGTGCGGCTCTTCACCAAGCATGAGTTATTTGCTGGTATAAAGG
ATGGGGAAAGCCTGCAGCTCTTTGAACAAAGTTCTCGCTCTGCGCATAAAC
AAGAGACACACACCATGGAGGCCGTGAAGCTTGTTGAGTTTGATCTTAAGC
AAACGCTTACCAGGCTCATGGCACATCTTTTTGGAGATGAGCCGGAGATAA
GGTGGGTAGACTGCTACTTCCCTTTTGGACATCCTTCCTTTGAGATGGAGAT
CAACTTTCATGGAGAATGGCTGGAAGTTCTTGGCTGCGGGGTGTTGGAACA
ACAACTGGTCAATTCAGCTGGTGCTCAAGACCGAATCGGCTGGGGATTTGG
CCTAGGGTTAGAAAGGCTAGCCATGATCCTCTACGACATCCCTGATATCCGT
CTCTTCTGGTGTGAGGACGAGCGCTTCCTGAAGCAGTTCTGTGTATCCAACA
TTAATCAGAAGGTGAAGTTTCAGCCTCTTAGCAAA

12D4-AzFRS-1 protein sequence

MDKKPLDVLISATGLWMSRTGTLHKIKHYEISRSKIYIEMACGDHLVVNNSRSC
RPARAFRYHKYRKTCKRCRVSDEDINNFLTRSTEGKTSVKVKVVSEPKVKKAM
PKSVSRAPKPLENPVSAKASTDTSRSVPSPAKSTPNSPVPTSASAPALTKSQTDR
LEVLLNPKDEISLNSGKPFRELESELLSRRKKDLQQIYAEEREGGGSGGGSQAW
GSRPPAAECATQRAPGSVVELLGKSYPQDDHSNLTRKVLTRVGRNLHNQQHHP
LWLIKERVLEHFNKQYVGSSGTPLFSVYDNLSPVVTTWQNFDSLLIPADHPCRK
KGDNYYLNRTHMLRAHTSAHQWDLLHAGLDAFLVVGDVYRRDQIDSQHYPIF
HQLEAVRLFTKHELFAGIKDGESLQLFEQSSRSAHKQETHTMEAVKLVEFDLK
QTLTRLMAHLFGDEPEIRWVDCYFPFGHPSFEMEINFHGEWLEVLGCGVLEQQ
LVNSAGAQDRIGWGFGLGLERLAMILYDIPDIRLFWCEDERFLKQFCVSNINQK
VKFQPLSK

13E3-AzFRS-1 DNA sequence

ATGGATAAGAAGCCGCTGGATGTTCTGATCTCTGCGACCGGTCTGTGGATGT
CCCGTACCGGCACGCTGCACAAGATCAAGCACTATGAGATTTCTCGTTCTAA
AATCTACATCGAAATGGCGTGTGGTGACCATCTGGTTGTGAACAACTCTCGT
TCTTGTCGTCCCGCACGTGCATTCCGTTATCATAAATACCGTAAAACCTGCA
AACGTTGTCGTGTTTCTGACGAAGATATCAACAACTTCCTGACCCGTTCTAC
CGAAGGCAAAACCTCTGTTAAAGTTAAAGTTGTTTCTGAGCCGAAAGTGAA
AAAAGCGATGCCGAAATCTGTTTCTCGTGCGCCGAAACCGCTGGAAAATCC
GGTTTCTGCGAAAGCGTCTACCGACACCTCTCGTTCTGTTCCGTCTCCGGCG
AAATCTACCCCGAACTCTCCGGTTCCGACCTCTGCAAGCGCCCCAGCTCTGA
CTAAATCCCAGACGGACCGTCTGGAGGTGCTGCTGAACCCAAAGGATGAAA
TCTCTCTGAACAGCGGCAAGCCTTTCCGTGAGCTGGAAAGCGAGCTGCTGTC
TCGTCGTAAAAAGGATCTGCAACAGATCTACGCTGAGGAACGCGAGGGTGG
CGGAAGCGGCGGCGGAAGCGGTGGCGGAAGTGGTGGCGGAAGCGGCGGCG
GAAGCCAGGCCTGGGGATCGAGGCCTCCTGCAGCAGAGTGTGCCACCCAAA
GAGCTCCAGGCAGTGTGGTGGAGCTGCTGGGCAAATCCTACCCTCAGGACG
ACCACAGCAACCTCACCCGGAAGGTCCTCACCAGAGTTGGCAGGAACCTGC
ACAACCAGCAGCATCACCCTCTGTGGCTGATCAAGGAGAGGGTGAAGGAGC
ACTTCAACAAGCAGTATGTGGACTGCTTTGGGACCCCGTTGTTCTCGGTCTA
CGACAACCTTTCTCCAGTGGTCACGACCTGGCAGAACTTTGACAGCCTGCTC
ATCACAGCTGATCACCCCTGCAGGAGGAAGGGGGACAACTATTACCTGAAT
CGGACTCACATGCTGAGAGCGCACACGTCCGCACACCAGTGGGACTTGCTG
CACGCGGGACTGGATGCCTTCCTGGTGGTGGGTGATGTCTACAGGCGAGAC
CAGATCGACTCCCAGCACTACCCTATTTTCCACCAGCTGGAGGCCGTGCGGC
TCTTCACCAAGCATGAGTTATTTGCTGGTATAAAGGATGGAGAAAGCCAGC
AGCTCTTTGAACAAAGTTCTCGCTCTGCGCATAAACAAGAGTCACACACCAT
GGAGGCCGTGAAGCTTGTAGAGTTTGATCTTAAGCAAACGCTTACCAGGCT
CATGGCACATCTTTTTGGAGATGAGCCGGAGATAAGATGGGTAGACAGCTA

CTTCCCTTTTGGACATCCTTCCTTTGAGATGGAGATCAACTTTCATGGAGAA
TGGCTGGAAGTTCTTGGCTGCGGGGTGTTGGAACAACAACTGGTCAATTCA
GCTGGTGCTCAAGACCGAATCGGCTGGGGATTCGGCCTAGGATTAGAAAGG
CTGGCCATGATCCTCTACGACATCCCTGATATCCGTCTCTTCTGGTGTGAGG
ACGAGCGCTTCCTGAAGCAGTTCTGTGTATCCAACATTAATCAGAAGGTGA
AGTTTCAGCCTCTTAGCAAA

13E3-AzFRS-1 protein sequence

MDKKPLDVLISATGLWMSRTGTLHKIKHYEISRSKIYIEMACGDHLVVNNSRSC
RPARAFRYHKYRKTCKRCRVSDEDINNFLTRSTEGKTSVKVKVVSEPKVKKAM
PKSVSRAPKPLENPVSAKASTDTSRSVPSPAKSTPNSPVPTSASAPALTKSQTDR
LEVLLNPKDEISLNSGKPFRELESELLSRRKKDLQQIYAEEREGGGSGGGSGGGS
GGGSGGGSQAWGSRPPAAECATQRAPGSVVELLGKSYPQDDHSNLTRKVLTR
VGRNLHNQQHHPLWLIKERVKEHFNKQYVDCFGTPLFSVYDNLSPVVTTWQN
FDSLLITADHPCRRKGDNYYLNRTHMLRAHTSAHQWDLLHAGLDAFLVVGDV
YRRDQIDSQHYPIFHQLEAVRLFTKHELFAGIKDGESQQLFEQSSRSAHKQESHT
MEAVKLVEFDLKQTLTRLMAHLFGDEPEIRWVDSYFPFGHPSFEMEINFHGEW
LEVLGCGVLEQQLVNSAGAQDRIGWGFGLGLERLAMILYDIPDIRLFWCEDERF
LKQFCVSNINQKVKFQPLSK

Supplementary Sequence 6. The plasmid maps and sequences

The gene of *dnaN* is underlined with solid line, synthetic sequence for amber codon incorporation is underlined with dash line, chPheT is underlined with dot line, and chPheRS is underlined with wavy line.

Vector: pChira-tac-2*TAG-dnaN-oxb20-chPheRS-2*chPheT

GTTGGCACTGATGAGGGTGTCAGTGAAGTGCTTCATGTGGCAGGAGAAAAA

AGGCTGCACCGGTGCGTCAGCAGAATATGTGATACAGGATATATTCCGCTTCC
TCGCTCACTGACTCGCTACGCTCGGTCGTTCGACTGCGGCGAGCGGAAATGG
CTTACGAACGGGGCGGAGATTTCCTGGAAGATGCCAGGAAGATACTTAACAG
GGAAGTGAGAGGGCCGCGGCAAAGCCGTTTTTCCATAGGCTCCGCCCCCCTG
ACAAGCATCACGAAATCTGACGCTCAAATCAGTGGTGGCGAAACCCGACAG
GACTATAAAGATACCAGGCGTTTCCCCCTGGCGGCTCCCTCGTGCGCTCTCCT
GTTCCTGCCTTTCGGTTTACCGGTGTCATTCCGCTGTTATGGCCGCGTTTGTCT
CATTCCACGCCTGACACTCAGTTCCGGGTAGGCAGTTCGCTCCAAGCTGGAC
TGTATGCACGAACCCCCCGTTCAGTCCGACCGCTGCGCCTTATCCGGTAACTA
TCGTCTTGAGTCCAACCCGGAAAGACATGCAAAAGCACCACTGGCAGCAGC
CACTGGTAATTGATTTAGAGGAGTTAGTCTTGAAGTCATGCGCCGGTTAAGGC
TAAACTGAAAGGACAAGTTTTGGTGACTGCGCTCCTCCAAGCCAGTTACCTC
GGTTCAAAGAGTTGGTAGCTCAGAGAACCTTCGAAAAACCGCCCTGCAAGG
CGGTTTTTTCGTTTTCAGAGCAAGAGATTACGCGCAGACCAAAACGATCTCA
AGAAGATCATCTTATTAATCAGATAAAATATTTCTAGATTTCAGTGCAATTTATC
TCTTCAAATGTAGCACCTGAAGTCAGCTAACAATGCGCTCATCGTCATCCTCG
GCACCGTCACCCTGGATGCTGTAGGCATAGGCTTGGTTATGCCGGTACTGCCG
GGCTTGACAATTAATCATCGGCTCGTATAATGCATACCCGTTTTTTTGGGCTAG
AAATAATTTTGTTTAACTTTAAGAAGGAGATATACATATGTAGGTTTAGGGTTA
GGGTGGTAAATTTACCGTAGAACGTGAGCATTTATTAAAACCGCTACAACAG
GTGAGCGGTCCGTTAGGTGGTCGTCCTACGCTACCGATTCTCGGTAATCTGCT
GTTACAGGTTGCTGACGGTACGTTGTCGCTGACCGGTACTGATCTCGAGATG
GAAATGGTGGCACGTGTTGCGCTGGTTCAGCCACACGAGCCAGGAGCGACG
ACCGTTCCGGCGCGCAAATTCTTTGATATCTGCCGTGGTCTGCCTGAAGGCGC
GGAAATTGCCGTGCAGCTGGAAGGTGAACGGATGCTGGTACGCTCCGGGCG
TAGCCGTTTTTCGCTGTCTACCCTGCCAGCGGCGGATTTCCCGAACCTCGATG
ACTGGCAGAGTGAAGTCGAATTTACCCTGCCGCAGGCAACGATGAAGCGTCT

GATTGAAGCGACCCAGTTTTCTATGGCGCATCAGGACGTTCGCTATTACTTAA

ATGGTATGCTGTTTGAAACCGAAGGTGAAGAACTGCGCACCGTGGCAACCG

ACGGCCACCGTCTGGCGGTCTGTTCAATGCCAATTGGTCAATCTTTGCCAAG

CCATTCGGTGATCGTACCGCGTAAAGGCGTGATTGAACTGATGCGTATGCTCG

ACGGCGGCGACAATCCGCTGCGCGTACAGATTGGCAGCAACAACATTCGCGC

CCACGTTGGCGACTTTATCTTCACCTCCAAACTGGTGGATGGTCGCTTCCCGG

ATTATCGCCGCGTTCTGCCGAAGAACCCGGACAAACATCTGGAAGCTGGCTG

CGATCTGCTCAAGCAGGCGTTTGCTCGCGCGGCGATTCTCTCTAACGAGAAA

TTCCGCGGCGTACGTCTTTATGTCAGCGAAAACCAGCTGAAAATCACCGCCA

ACAACCCGGAACAGGAAGAAGCGGAAGAGATCCTCGACGTTACCTATAGCG

GTGCGGAGATGGAAATCGGCTTCAACGTCAGTTATGTGCTGGATGTTCTGAA

CGCGCTGAAATGCGAAAACGTCCGCATGATGCTGACCGATTCGGTTTCCAGC

GTGCAGATTGAAGATGCGGCCAGCCAGAGCGCGGCTTATGTTGTCATGCCAA

TGAGACTGCATCATCACCATCACCATTAAGCATGCACCATTCCTTGCGGCGGC

GGTGCTCAACGGCCTCAACCTACTACTGGGCTGCTTCCTAATGCAGGAGTCG

CATAAGGGAGAGCGTCTGGCGAAAGGGGGATGTGCTGCAAGGCGATTAAGT

TGGGTAACGCCAGGGTTTTCCCAGTCACGACGTTGTAAAACGACGGCCAGT

GCCAAGCTTAAAAAAAATCCTTAGCTTTCGCTAAGGATCTGCAGTGGTGTGA

GAGCCGGGAATCTAACCCGGCTGAACGGATTTAGAGTCCGTTCGATCTACAT

GATCACTCTCACGAATTCAGCGTTACAAGTATTACACAAAGTTTTTATGTTG

AGAATATTTTTTGATGGGACTAGTTAAAAAAAATCCTTAGCTTTCGCTAAGG

ATCTGCAGTGGTGTGAGAGCCGGGAATCTAACCCGGCTGAACGGATTTAGAG

TCCGTTCGATCTACATGATCACTCTCACGAATTCAGCGTTACAAGTATTACAC

AAAGTTTTTATGTTGAGAATATTTTTTGATGGGGCGCCACTTATTTTTGATC

GTTCGCTCAAAGAAGCGGCGCCAGGGTTGTTTTTCTTTTCACCAGTGAGACG

GGCAACAGAACGCCATAGCGGCCTCATTTCTTATTCTGAGTTACAACAGTCCG

CACCGCTGCCGGTAGCTCCTTCCGGTGGGCGCGGGGCATGACTATCGTCGCC

GCACTTATGACTGTCTTCTTTATCATGCAACTCGTAGGACAGGTGCCGGCAGC

GCCCAACAGTCCCCCGGCCACGGGGCCTGCCACCATACCCACGCCGAAACA

AGCGCCCTGCACCATTATGTTCCGGATCTGCATCGCAGGATGCTGCTGGCTAC

CCTGTGGAACACCTACATCTGTATTAACGAAGCGCTAACCGTTTTTATCATGC

TCTGGGAGGCAGAATAAATGGCTGAGTTGAAGGATCCTCGGGTTGTCAGCCT

GTCCCGCTTATAAAAGCTGTTGTGACCGCTTGCTCTAGCCAGCTATCGAGTTG

TGAACCGATCCATCTAGCAATTGGTCTCGATCTAGCGATAGGCTTCGATCTAG

CTATGTAGAAACGCCGTGTGCTCGATCGCTTGATAAGGTCCACGTAGCTGCTA

TAATTGCTTCAACAGAACATATTGACTATCCGGTATTACCCGGCCGCCGTTATA

CGTTGTTTACGCTTTGAGGAATCCCATATGGATAAGAAGCCGCTGGATGTTCT

GATCTCTGCGACCGGTCTGTGGATGTCCCGTACCGGCACGCTGCACAAGATC

AAGCACTATGAGATTTCTCGTTCTAAAATCTACATCGAAATGGCGTGTGGTGA

CCATCTGGTTGTGAACAACTCTCGTTCTTGTCGTCCCGCACGTGCATTCCGTT

ATCATAAATACCGTAAAACCTGCAAACGTTGTCGTGTTTCTGACGAAGATATC

AACAACTTCCTGACCCGTTCTACCGAAGGCAAAACCTCTGTTAAAGTTAAAG

TTGTTTCTGAGCCGAAAGTGAAAAAAGCGATGCCGAAATCTGTTTCTCGTGC

GCCGAAACCGCTGGAAAATCCGGTTTCTGCGAAAGCGTCTACCGACACCTCT

CGTTCTGTTCCGTCTCCGGCGAAATCTACCCCGAACTCTCCGGTTCCGACCTC

TGCAAGCGCCCCAGCTCTGACTAAATCCCAGACGGACCGTCTGGAGGTGCTG

CTGAACCCAAAGGATGAAATCTCTCTGAACAGCGGCAAGCCTTTCCGTGAGC

TGGAAAGCGAGCTGCTGTCTCGTCGTAAAAAGGATCTGCAACAGATCTACGC

TGAGGAACGCGAGGGTGGCGGAAGCGGCGGCGGAAGCGGTGGCGGAAGTG

GTGGCGGAAGCGGCGGCGGAAGCCAGGCCTGGGGATCGAGGCCTCCTGCAG

CAGAGTGTGCCACCCAAAGAGCTCCAGGCAGTGTGGTGGAGCTGCTGGGCA

AATCCTACCCTCAGGACGACCACAGCAACCTCACCCGGAAGGTCCTCACCA

GAGTTGGCAGGAACCTGCACAACCAGCAGCATCACCCTCTGTGGCTGATCA

AGGAGAGGGTGAAGGAGCACTTCTACAAGCAGTATGTGGGCCGCTTTGGGA

CCCCGTTGTTCTCGGTCTACGACAACCTTTCTCCAGTGGTCACGACCTGGCA
GAACTTTGACAGCCTGCTCATCCCAGCTGATCACCCCAGCAGGAAGAAGGG
GGACAACTATTACCTGAATCGGACTCACATGCTGAGATCGCACACGTCTGCA
CACcagTGGGACTTGCTGCACGCGGGACTGGATGCCTTCCTGGTGGTGGGTGA
TGTCTACAGGCGTGACCAGATCGACTCCCAGCACTACCCTATTTTCCACCAGC
TGGAGGCCGTGCGGCTCTTCTCCAAGCATGAGTTATTTGCTGGTATAAAGGAT
GGAGAAAGCCTGCAGCTCTTTGAACAAAGTTCTCGCTCTGCGCATAAACAAG
AGACACACACCATGGAGGCCGTGAAGCTTGTAGAGTTTGATCTTAAGCAAAC
GCTTACCAGGCTCATGGCACATCTTTTTGGAGATGAGCTGGAGATAAGATGG
GTAGACTGCTACATTCCTTTtGGtCATCCTTCgTTTGAGATGGAGATCAACTTTC
ATGGAGAATGGCTGGAAGTTCTTGGCTGCGGGGTGatgGAACAACAACTGGT
CAATTCAGCTGGTGCTCAAGACCGAATCGGCTGGGGATTTGGCCTAGGATTA
GAAAGGCTAGCCATGATCCTCTACGACATCCCTGATATCCGTCTCTTCTGGTG
TGAGGACGAGCGCTTCCTGAAGCAGTTCTGTGTATCCAACATTAATCAGAAG
GTGAAGTTTCAGCCTCTTAGCAAATAACTGCAGTTTCAAACGCTAAATTGCCT
GATGCGCTACGCTTATCAGGCCTACATGATCTCTGCAATATATTGAGTTTGCGT
GCTTTTGTAGGCCGGATAAGGCGTTCACGCCGCATCCGGCAAGAAACAGCAA
ACAATCCAAAACGCCGCGTTCAGCGGCGTTTTTCTGCTTTTCTTCGCGAATT
AATTCCGCTTCGCACATGTGAGCAAAAGGCCAGCAAAAGGCCAGATCATATC
GTCAATTATTACCTCCACGGGGAGAGCCTGAGCAAACTGGCCTCAGGCATTT
GAGAAGCACACGGTCACACTGCTTCCGGTAGTCAATAAACCGGTAAACCAG
CAATAGACATAAGCGGCTATTTAACGACCCTGCCCTGAACCGACGACCGGGT
CGAATTTGCTTTCGAATTTCTGCCATTCATCCGCTTATTATCACTTATTCAGGC
GTAGCAACCAGGCGTTTAAGGGCACCAATAACTGCCTTAAAAAAATTACGCC
CCGCCCTGCCACTCATCGCAGTACTGTTGTAATTCATTAAGCATTCTGCCGAC
ATGGAAGCCATCACAAACGGCATGATGAACCTGAATCGCGAATTTGCTTTCG
AATTTCTGCCATTCATCCGCTTATTATCACTTATTCAGGCGTAGCAACCAGGCG

TTTAAGGGCACCAATAACTGCCTTAAAAAAATTACGCCCCGCCCTGCCACTC
ATCGCAGTACTGTTGTAATTCATTAAGCATTCTGCCGACATGGAAGCCATCAC
AAACGGCATGATGAACCTGAATCGCCAGCGGCATCAGCACCTTGTCGCCTTG
CGTATAATATTTGCCCATGGTGAAAACGGGGGCGAAGAAGTTGTCCATATTGG
CCACGTTTAAATCAAAACTGGTGAAACTCACCCAGGGATTGGCTGAGACGA
AAAACATATTCTCAATAAACCCTTTAGGGAAATAGGCCAGGTTTTCACCGTAA
CACGCCACATCTTGCGAATATATGTGTAGAAACTGCCGGAAATCGTCGTGGTA
TTCACTCCAGAGCGATGAAAACGTTTCAGTTTGCTCATGGAAAACGGTGTAA
CAAGGGTGAACACTATCCCATATCACCAGCTCACCGTCTTTCATTGCCATACG
GAATTCCGGATGAGCATTCATCAGGCGGGCAAGAATGTGAATAAAGGCCGGA
TAAAACTTGTGCTTATTTTTCTTTACGGTCTTTAAAAAGGCCGTAATATCCAGC
TGAACGGTCTGGTTATAGGTACATTGAGCAACTGACTGAAATGCCTCAAAAT
GTTCTTTACGATGCCATTGGGATATATCAACGGTGGTATATCCAGTGATTTTTTT
CTCCATTTTAGCTTCCTTAGCTCCTGAAAATCTCGATAACTCAAAAAATACGC
CCGGTAGTGATCTTATTTCATTATGGTGAAAGTTGGAACCTCTTACGTGCCGA
TCAACGTCTCATTTTCGCCAAAAGTTGGCCCAGGGCTTCCCGGTATCAACAG
GGACACCAGGATTTATTTATTCTGCGAAGTGATCTTCCGTCACAGGTATTTATT
CGGCGCAAAGTGCGTCGGGTGATGCTGCCAACTTACTGATTTAGTGTATGATG
GTGTTTTTGAGGTGCTCCAGTGGCTTCTGTTTCTATCAGCTGTCCCTCCTGTT
CAGCTACTGACGGGGTGGTGCGTAACGGCAAAAGCACCGCCGGACATCAGC
GCTAGCGGAGTGTATACTGGCTTACTAT

Supplementary Sequence 7. The DNA sequences of proteins for amber suppression assay

The amber codon for UAA incorporation is highlighted in yellow.

UBE2K-D48TAG

ATGGCCAACATCGCGGTGCAGCGAATCAAGCGGGAGTTCAAGGAGGTGCTG
AAGAGCGAGGAGACGAGCAAAAATCAAATTAAAGTAGATCTTGTAGATGA
GAATTTTACAGAATTAAGAGGAGAAATAGCAGGACCTCCATAGACACCATA
TGAAGGAGGAAGATACCAACTAGAGATAAAAATACCAGAAACATACCCAT
TTAATCCCCCTAAGGTCCGGTTTATCACTAAAATATGGCATCCTAATATTAG
TTCCGTCACAGGGGCTATTTGTTTGGATATCCTGAAAGATCAATGGGCAGCT
GCAATGACTCTCCGCACGGTATTATTGTCATTGCAAGCACTATTGGCAGCTG
CAGAGCCAGATGATCCACAGGATGCTGTAGTAGCAAATCAGTACAAACAAA
ATCCCGAAATGTTCAAACAGACAGCTCGACTTTGGGCACATGTGTATGCTG
GAGCACCAGTTTCTAGTCCAGAATACACCAAAAAAATAGAAAACCTATGTG
CTATGGGCTTTGATAGGAATGCAGTAATAGTGGCCTTGTCTTCAAAATCATG
GGATGTAGAGACTGCAACAGAATTGCTTCTGAGTAAC

UBE2K-Y134TAG

ATGGCCAACATCGCGGTGCAGCGAATCAAGCGGGAGTTCAAGGAGGTGCTG
AAGAGCGAGGAGACGAGCAAAAATCAAATTAAAGTAGATCTTGTAGATGA
GAATTTTACAGAATTAAGAGGAGAAATAGCAGGACCTCCAGACACACCATA
TGAAGGAGGAAGATACCAACTAGAGATAAAAATACCAGAAACATACCCAT
TTAATCCCCCTAAGGTCCGGTTTATCACTAAAATATGGCATCCTAATATTAG
TTCCGTCACAGGGGCTATTTGTTTGGATATCCTGAAAGATCAATGGGCAGCT
GCAATGACTCTCCGCACGGTATTATTGTCATTGCAAGCACTATTGGCAGCTG
CAGAGCCAGATGATCCACAGGATGCTGTAGTAGCAAATCAGTAGAAACAAA
ATCCCGAAATGTTCAAACAGACAGCTCGACTTTGGGCACATGTGTATGCTG
GAGCACCAGTTTCTAGTCCAGAATACACCAAAAAAATAGAAAACCTATGTG
CTATGGGCTTTGATAGGAATGCAGTAATAGTGGCCTTGTCTTCAAAATCATG
GGATGTAGAGACTGCAACAGAATTGCTTCTGAGTAAC

ADK-E114TAG

ATGCGTATCATTCTGCTTGGCGCTCCGGGCGCGGGGAAAGGGACTCAGGCT
CAGTTCATCATGGAGAAATATGGTATTCCGCAAATCTCCACTGGCGATATGC
TGCGTGCTGCGGTCAAATCTGGCTCCGAGCTGGGTAAACAAGCAAAAGACA
TTATGGATGCTGGCAAACTGGTCACCGACGAACTGGTGATCGCGCTGGTTA
AAGAGCGCATTGCTCAGGAAGACTGCCGTAATGGTTTCCTGTTGGACGGCTT
CCCGCGTACCATTCCGCAGGCAGACGCGATGAAAGAAGCGGGCATCAATGT
TGATTACGTTCTGGAATTCGACGTACCGGACTAGCTGATCGTTGACCGTATC
GTCGGTCGCCGCGTTCATGCGCCGTCTGGTCGTGTTTATCACGTTAAATTCA
ATCCGCCGAAAGTCGAAGGCAAAGACGACGTTACCGGTGAAGAACTGACTA
CCCGTAAAGATGATCAGGAAGAGACCGTACGTAAACGTCTGGTTGAATACC
ATCAGATGACAGCACCGCTGATCGGCTACTACTCCAAAGAAGCAGAAGCGG
GTAATACCAAATACGCGAAAGTTGACGGCACCAAGCCGGTTGCTGAAGTTC
GCGCTGATCTGGAAAAAATCCTCGGC

ADK-V142TAG

ATGCGTATCATTCTGCTTGGCGCTCCGGGCGCGGGGAAAGGGACTCAGGCT
CAGTTCATCATGGAGAAATATGGTATTCCGCAAATCTCCACTGGCGATATGC
TGCGTGCTGCGGTCAAATCTGGCTCCGAGCTGGGTAAACAAGCAAAAGACA
TTATGGATGCTGGCAAACTGGTCACCGACGAACTGGTGATCGCGCTGGTTA
AAGAGCGCATTGCTCAGGAAGACTGCCGTAATGGTTTCCTGTTGGACGGCTT
CCCGCGTACCATTCCGCAGGCAGACGCGATGAAAGAAGCGGGCATCAATGT
TGATTACGTTCTGGAATTCGACGTACCGGACGAGCTGATCGTTGACCGTATC
GTCGGTCGCCGCGTTCATGCGCCGTCTGGTCGTGTTTATCACGTTAAATTCA
ATCCGCCGAAATAGGAAGGCAAAGACGACGTTACCGGTGAAGAACTGACT
ACCCGTAAAGATGATCAGGAAGAGACCGTACGTAAACGTCTGGTTGAATAC
CATCAGATGACAGCACCGCTGATCGGCTACTACTCCAAAGAAGCAGAAGCG

GGTAATACCAAATACGCGAAAGTTGACGGCACCAAGCCGGTTGCTGAAGTT
CGCGCTGATCTGGAAAAAATCCTCGGC

TyrRS-E240TAG

ATGGCAAGCAGTAACTTGATTAAACAATTGCAAGAGCGGGGGCTGGTAGCC
CAGGTGACGGACGAGGAAGCGTTAGCAGAGCGACTGGCGCAAGGCCCGAT
CGCGCTCTATTGCGGCTTCGATCCTACCGCTGACAGCTTGCATTTGGGGCAT
CTTGTTCCATTGTTATGCCTGAAACGCTTCCAGCAGGCGGGCCACAAGCCGG
TTGCGCTGGTAGGCGGCGCGACGGGTCTGATTGGCGACCCGAGCTTCAAAG
CTGCCGAGCGTAAGCTGAACACCGAAGAAACTGTTCAGGAGTGGGTGGACA
AAATCCGTAAGCAGGTTGCCCCGTTCCTCGATTTCGACTGTGGAGAAAACTC
TGCTATCGCGGCGAACAACTATGACTGGTTCGGCAATATGAATGTGCTGAC
CTTCCTGCGCGATATTGGCAAACACTTCTCCGTTAACCAGATGATCAACAAA
GAAGCGGTTAAGCAGCGTCTCAACCGTGAAGATCAGGGGATTTCGTTCACT
GAGTTTTCCTACAACCTGTTGCAGGGTTATGACTTCGCCTGTCTGAACAAAC
AGTACGGTGTGGTGCTGCAAATTGGTGGTTCTGACCAGTGGGGTAACATCA
CTTCTGGTATCGACCTGACCCGTCGTCTGCATCAGAATCAGGTGTTTGGCCT
GACCGTTCCGCTGATCACTAAAGCAGATGGCACCAAATTTGGTAAAACTTA
GGGCGGCGCAGTCTGGTTGGATCCGAAGAAAACCAGCCCGTACAAATTCTA
CCAGTTCTGGATCAACACTGCGGATGCCGACGTTTACCGCTTCCTGAAGTTC
TTCACCTTTATGAGCATTGAAGAGATCAACGCCCTGGAAGAAGAAGATAAA
AACAGCGGTAAAGCACCGCGCGCCCAGTATGTACTGGCGGAGCAGGTGACT
CGTCTGGTTCACGGTGAAGAAGGTTTACAGGCGGCAAAACGTATTACCGAA
TGCCTGTTCAGCGGTTCTTTGAGTGCGCTGAGTGAAGCGGACTTCGAACAGC
TGGCGCAGGACGGCGTACCGATGGTTGAGATGGAAAAGGGCGCAGACCTG
ATGCAGGCACTGGTCGATTCTGAACTGCAACCTTCCCGTGGTCAGGCACGTA
AAACTATCGCCTCCAATGCCATCACCATTAACGGTGAAAAACAGTCCGATC

CTGAATACTTCTTTAAAGAAGAAGATCGTCTGTTTGGTCGTTTTACCTTACT
GCGTCGCGGTAAAAAGAATTACTGTCTGATTTGCTGGAAA

TyrRS-Y301TAG

ATGGCAAGCAGTAACTTGATTAAACAATTGCAAGAGCGGGGGCTGGTAGCC
CAGGTGACGGACGAGGAAGCGTTAGCAGAGCGACTGGCGCAAGGCCCGAT
CGCGCTCTATTGCGGCTTCGATCCTACCGCTGACAGCTTGCATTTGGGGCAT
CTTGTTCCATTGTTATGCCTGAAACGCTTCCAGCAGGCGGGCCACAAGCCGG
TTGCGCTGGTAGGCGGCGCGACGGGTCTGATTGGCGACCCGAGCTTCAAAG
CTGCCGAGCGTAAGCTGAACACCGAAGAAACTGTTCAGGAGTGGGTGGACA
AAATCCGTAAGCAGGTTGCCCCGTTCCTCGATTTCGACTGTGGAGAAAACTC
TGCTATCGCGGCGAACAACTATGACTGGTTCGGCAATATGAATGTGCTGAC
CTTCCTGCGCGATATTGGCAAACACTTCTCCGTTAACCAGATGATCAACAAA
GAAGCGGTTAAGCAGCGTCTCAACCGTGAAGATCAGGGGATTTCGTTCACT
GAGTTTTCCTACAACCTGTTGCAGGGTTATGACTTCGCCTGTCTGAACAAAC
AGTACGGTGTGGTGCTGCAAATTGGTGGTTCTGACCAGTGGGGTAACATCA
CTTCTGGTATCGACCTGACCCGTCGTCTGCATCAGAATCAGGTGTTTGGCCT
GACCGTTCCGCTGATCACTAAAGCAGATGGCACCAAATTTGGTAAAACTGA
AGGCGGCGCAGTCTGGTTGGATCCGAAGAAAACCAGCCCGTACAAATTCTA
CCAGTTCTGGATCAACACTGCGGATGCCGACGTTTACCGCTTCCTGAAGTTC
TTCACCTTTATGAGCATTGAAGAGATCAACGCCCTGGAAGAAGAAGATAAA
AACAGCGGTAAAGCACCGCGCGCCCAGTAGGTACTGGCGGAGCAGGTGACT
CGTCTGGTTCACGGTGAAGAAGGTTTACAGGCGGCAAAACGTATTACCGAA
TGCCTGTTCAGCGGTTCTTTGAGTGCGCTGAGTGAAGCGGACTTCGAACAGC
TGGCGCAGGACGGCGTACCGATGGTTGAGATGGAAAAGGGCGCAGACCTG
ATGCAGGCACTGGTCGATTCTGAACTGCAACCTTCCCGTGGTCAGGCACGTA
AAACTATCGCCTCCAATGCCATCACCATTAACGGTGAAAAACAGTCCGATC

CTGAATACTTCTTTAAAGAAGAAGATCGTCTGTTTGGTCGTTTTACCTTACT
GCGTCGCGGTAAAAAGAATTACTGTCTGATTTGCTGGAAA

Fluc-D234TAG

ATGGAAGATGCCAAAAACATTAAGAAGGGCCCAGCGCCATTCTACCCACTC
GAAGACGGGACCGCCGGCGAGCAGCTGCACAAAGCCATGAAGCGCTACGC
CCTGGTGCCCGGCACCATCGCCTTTACCGACGCACATATCGAGGTGGACATT
ACCTACGCCGAGTACTTCGAGATGAGCGTTCGGCTGGCAGAAGCTATGAAG
CGCTATGGGCTGAATACAAACCATCGGATCGTGGTGTGCAGCGAGAATAGC
TTGCAGTTCTTCATGCCCGTGTTGGGTGCCCTGTTCATCGGTGTGGCTGTGG
CCCCAGCTAACGACATCTACAACGAGCGCGAGCTGCTGAACAGCATGGGCA
TCAGCCAGCCCACCGTCGTATTCGTGAGCAAGAAAGGGCTGCAAAAGATCC
TCAACGTGCAAAAGAAGCTACCGATCATACAAAAGATCATCATCATGGATA
GCAAGACCGACTACCAGGGCTTCCAAAGCATGTACACCTTCGTGACTTCCC
ATTTGCCACCCGGCTTCAACGAGTACGACTTCGTGCCCGAGAGCTTCGACCG
GGACAAAACCATCGCCCTGATCATGAACAGTAGTGGCAGTACCGGATTGCC
CAAGGGCGTAGCCCTACCGCACCGCACCGCTTGTGTCCGATTCAGTCATGCC
CGCGACCCCATCTTCGGCAACCAGATCATCCCCTAGACCGCTATCCTCAGCG
TGGTGCCATTTCACCACGGCTTCGGCATGTTCACCACGCTGGGCTACTTGAT
CTGCGGCTTTCGGGTCGTGCTCATGTACCGCTTCGAGGAGGAGCTATTCTTG
CGCAGCTTGCAAGACTATAAGATTCAATCTGCCCTGCTGGTGCCCACACTAT
TTAGCTTCTTCGCTAAGAGCACTCTCATCGACAAGTACGACCTAAGCAACTT
GCACGAGATCGCCAGCGGCGGGGCGCCGCTCAGCAAGGAGGTAGGTGAGG
CCGTGGCCAAACGCTTCCACCTACCAGGCATCCGCCAGGGCTACGGCCTGA
CAGAAACAACCAGCGCCATTCTGATCACCCCCGAAGGGGACGACAAGCCTG
GCGCAGTAGGCAAGGTGGTGCCCTTCTTCGAGGCTAAGGTGGTGGACTTGG
ACACCGGTAAGACACTGGGTGTGAACCAGCGCGGCGAGCTGTGCGTCCGTG

GCCCCATGATCATGAGCGGCTACGTTAACAACCCCGAGGCTACAAACGCTC
TCATCGACAAGGACGGCTGGCTGCACAGCGGCGACATCGCCTACTGGGACG
AGGACGAGCACTTCTTCATCGTGGACCGGCTGAAGAGCCTGATCAAATACA
AGGGCTACCAGGTAGCCCCAGCCGAACTGGAGAGCATCCTGCTGCAACACC
CCAACATCTTCGACGCCGGGGTCGCCGGCCTGCCCGACGACGATGCCGGCG
AGCTGCCCGCCGCAGTCGTCGTGCTGGAACACGGTAAAACCATGACCGAGA
AGGAGATCGTGGACTATGTGGCCAGCCAGGTTACAACCGCCAAGAAGCTGC
GCGGTGGTGTTGTGTTCGTGGACGAGGTGCCTAAAGGACTGACCGGCAAGT
TGGACGCCCGCAAGATCCGCGAGATTCTCATTAAGGCCAAGAAGGGCGGCA
AGATCGCCGTG

Fluc-Y266TAG

ATGGAAGATGCCAAAAACATTAAGAAGGGCCCAGCGCCATTCTACCCACTC
GAAGACGGGACCGCCGGCGAGCAGCTGCACAAAGCCATGAAGCGCTACGC
CCTGGTGCCCGGCACCATCGCCTTTACCGACGCACATATCGAGGTGGACATT
ACCTACGCCGAGTACTTCGAGATGAGCGTTCGGCTGGCAGAAGCTATGAAG
CGCTATGGGCTGAATACAAACCATCGGATCGTGGTGTGCAGCGAGAATAGC
TTGCAGTTCTTCATGCCCGTGTTGGGTGCCCTGTTCATCGGTGTGGCTGTGG
CCCCAGCTAACGACATCTACAACGAGCGCGAGCTGCTGAACAGCATGGGCA
TCAGCCAGCCCACCGTCGTATTCGTGAGCAAGAAAGGGCTGCAAAAGATCC
TCAACGTGCAAAAGAAGCTACCGATCATACAAAAGATCATCATCATGGATA
GCAAGACCGACTACCAGGGCTTCCAAAGCATGTACACCTTCGTGACTTCCC
ATTTGCCACCCGGCTTCAACGAGTACGACTTCGTGCCCGAGAGCTTCGACCG
GGACAAAACCATCGCCCTGATCATGAACAGTAGTGGCAGTACCGGATTGCC
CAAGGGCGTAGCCCTACCGCACCGCACCGCTTGTGTCCGATTCAGTCATGCC
CGCGACCCCATCTTCGGCAACCAGATCATCCCCGACACCGCTATCCTCAGCG
TGGTGCCATTTCACCACGGCTTCGGCATGTTCACCACGCTGGGCTACTTGAT

CTGCGGCTTTCGGGTCGTGCTCATGTAGCGCTTCGAGGAGGAGCTATTCTTG
CGCAGCTTGCAAGACTATAAGATTCAATCTGCCCTGCTGGTGCCCACACTAT
TTAGCTTCTTCGCTAAGAGCACTCTCATCGACAAGTACGACCTAAGCAACTT
GCACGAGATCGCCAGCGGCGGGGCGCCGCTCAGCAAGGAGGTAGGTGAGG
CCGTGGCCAAACGCTTCCACCTACCAGGCATCCGCCAGGGCTACGGCCTGA
CAGAAACAACCAGCGCCATTCTGATCACCCCCGAAGGGGACGACAAGCCTG
GCGCAGTAGGCAAGGTGGTGCCCTTCTTCGAGGCTAAGGTGGTGGACTTGG
ACACCGGTAAGACACTGGGTGTGAACCAGCGCGGCGAGCTGTGCGTCCGTG
GCCCCATGATCATGAGCGGCTACGTTAACAACCCCGAGGCTACAAACGCTC
TCATCGACAAGGACGGCTGGCTGCACAGCGGCGACATCGCCTACTGGGACG
AGGACGAGCACTTCTTCATCGTGGACCGGCTGAAGAGCCTGATCAAATACA
AGGGCTACCAGGTAGCCCCAGCCGAACTGGAGAGCATCCTGCTGCAACACC
CCAACATCTTCGACGCCGGGGTCGCCGGCCTGCCCGACGACGATGCCGGCG
AGCTGCCCGCCGCAGTCGTCGTGCTGGAACACGGTAAAACCATGACCGAGA
AGGAGATCGTGGACTATGTGGCCAGCCAGGTTACAACCGCCAAGAAGCTGC
GCGGTGGTGTTGTGTTCGTGGACGAGGTGCCTAAAGGACTGACCGGCAAGT
TGGACGCCCGCAAGATCCGCGAGATTCTCATTAAGGCCAAGAAGGGCGGCA
AGATCGCCGTG

GID4-(116-300)-F128TAG

ATGGGCGTGGCCACCAGCCTGCTCTACAGCGGCTCCAAGTAGCGCGGCCAC
CAGAAGAGCAAGGGGAACTCGTACGACGTAGAGGTGGTGCTGCAGCACGT
GGACACGGGGAACTCTTACCTTTGTGGGTACTTGAAGATTAAAGGCCTTACT
GAGGAGTATCCAACCCTTACAACCTTCTTCGAAGGAGAAATAATCAGCAAA
AAACACCCTTTCTTAACTCGCAAGTGGGATGCAGATGAAGATGTTGATCGG
AAACACTGGGGCAAGTTTCTGGCTTTTTATCAGTATGCAAAATCATTTAACT
CAGATGACTTTGATTATGAAGAGCTGAAGAATGGAGACTACGTCTTCATGA

GGTGGAAGGAACAGTTTCTGGTCCCAGATCACACGATCAAAGACATCAGTG
GTGCTTCTTTTGCCGGGTTCTACTACATCTGCTTTCAGAAGTCAGCAGCCTCC
ATAGAGGGCTACTACTACCATAGGAGTTCAGAATGGTATCAGTCCCTCAAT
CTAACCCATGTTCCTGAACACAGTGCACCCATCTATGAATTCCGGGGTCATC
ATCACCATCACCATTGA

Neo2/15-Y14TAG

ATGGGCAGCAGCCATCATCATCATCATCACAGCAGCGGCCTGGTGCCGCGC
GGCAGCCCTAAAAAGAAAATCCAGCTGCACGCTGAACATGCACTGTAGGAT
GCACTGATGATCCTGAATATCGTCAAAACCAACAGCCCGCCGGCAGAAGAA
AAACTGGAAGATTATGCATTTAACTTTGAACTGATCCTGGAAGAAATTGCA
CGTCTGTTTGAAAGCGGTGATCAGAAAGATGAAGCAGAAAAAGCAAAACG
TATGAAAGAATGGATGAAACGCATTAAAACCACCGCAAGCGAAGATGAAC
AGGAAGAAATGGCAAATGCAATTATTACCATTCTGCAGAGCTGGATTTTA
GTTAA

Ub-S57TAG

ATGCAGATCTTCGTGAAGACTCTGACTGGTAAGACCATCACCCTCGAGGTG
GAGCCCAGTGACACCATCGAGAATGTCAAGGCAAAGATCCAAGATAAGGA
AGGCATTCCTCCTGATCAGCAGAGGTTGATCTTTGCCGGAAAACAGCTGGA
AGATGGTCGTACCCTGTAGGACTACAACATCCAGAAAGAGTCCACCTTGCA
CCTGGTGCTCCGTCTCAGAGGTGGGTGCGGTCATCATCACCATCACCATTGA

Ub-S65TAG

ATGCAGATCTTCGTGAAGACTCTGACTGGTAAGACCATCACCCTCGAGGTG
GAGCCCAGTGACACCATCGAGAATGTCAAGGCAAAGATCCAAGATAAGGA
AGGCATTCCTCCTGATCAGCAGAGGTTGATCTTTGCCGGAAAACAGCTGGA

AGATGGTCGTACCCTGTCTGACTACAACATCCAGAAAGAGTAGACCTTGCA
CCTGGTGCTCCGTCTCAGAGGTGGGTGCGGTCATCATCACCATCACCATTGA

BPTF(2583-2751)-GST-Y2587TAG

ATGGATACAAAACTGTAGTGCATCTGTAAAACGCCGTATGATGAAAGCAAA
TTTTATATTGGTTGCGATCGTTGTCAGAATTGGTATCATGGTCGTTGTGTGG
GTATTCTGCAGTCTGAAGCAGAACTGATTGATGAATATGTGTGTCCGCAGTG
TCAGAGCACCGAAGATGCGCTGGTGCCGCGCGGCAGCATGTCCCCTATACT
AGGTTATTGGAAAATTAAGGGCCTTGTGCAACCCACTCGACTTCTTTTGGAA
TATCTTGAAGAAAAATATGAAGAGCATTTGTATGAGCGCGATGAAGGTGAT
AAATGGCGAAACAAAAAGTTTGAATTGGGTTTGGAGTTTCCCAATCTTCCTT
ATTATATTGATGGTGATGTTAAATTAACACAGTCTATGGCCATCATACGTTA
TATAGCTGACAAGCACAACATGTTGGGTGGTTGTCCAAAAGAGCGTGCAGA
GATTTCAATGCTTGAAGGAGCGGTTTTGGATATTAGATACGGTGTTTCGAGA
ATTGCATATAGTAAAGACTTTGAAACTCTCAAAGTTGATTTTCTTAGCAAGC
TACCTGAAATGCTGAAAATGTTCGAAGATCGTTTATGTCATAAAACATATTT
AAATGGTGATCATGTAACCCATCCTGACTTCATGTTGTATGACGCTCTTGAT
GTTGTTTTATACATGGACCCAATGTGCCTGGATGCGTTCCCAAAATTAGTTT
GTTTTAAAAAACGTATTGAAGCTATCCCACAAATTGATAAGTACTTGAAATC
CAGCAAGTATATAGCATGGCCTTTGCAGGGCTGGCAAGCCACGTTTGGTGG
TGGCGACCATCCTCCAAAATGA

BPTF(2583-2751)-GST-W2609TAG

ATGGATACAAAACTGTATTGCATCTGTAAAACGCCGTATGATGAAAGCAAA
TTTTATATTGGTTGCGATCGTTGTCAGAATTAGTATCATGGTCGTTGTGTGG
GTATTCTGCAGTCTGAAGCAGAACTGATTGATGAATATGTGTGTCCGCAGTG
TCAGAGCACCGAAGATGCGATGCTGGTGCCGCGCGGCAGCATGTCCCCTAT

ACTAGGTTATTGGAAAATTAAGGGCCTTGTGCAACCCACTCGACTTCTTTG
GAATATCTTGAAGAAAAATATGAAGAGCATTTGTATGAGCGCGATGAAGGT
GATAAATGGCGAAACAAAAAGTTTGAATTGGGTTTGGAGTTTCCCAATCTTC
CTTATTATATTGATGGTGATGTTAAATTAACACAGTCTATGGCCATCATACG
TTATATAGCTGACAAGCACAACATGTTGGGTGGTTGTCCAAAAGAGCGTGC
AGAGATTTCAATGCTTGAAGGAGCGGTTTTGGATATTAGATACGGTGTTTCG
AGAATTGCATATAGTAAAGACTTTGAAACTCTCAAAGTTGATTTTCTTAGCA
AGCTACCTGAAATGCTGAAAATGTTCGAAGATCGTTTATGTCATAAAACAT
ATTTAAATGGTGATCATGTAACCCATCCTGACTTCATGTTGTATGACGCTCT
TGATGTTGTTTTATACATGGACCCAATGTGCCTGGATGCGTTCCCAAAATTA
GTTTGTTTTAAAAAACGTATTGAAGCTATCCCACAAATTGATAAGTACTTGA
AATCCAGCAAGTATATAGCATGGCCTTTGCAGGGCTGGCAAGCCACGTTTG
GTGGTGGCGACCATCCTCCAAAATGA

Supplementary Sequence 8. The table of PCR primers for DNA constructions and mutations

<table>
<tr><th>Primer</th><th>Sequence</th><th>Clone</th></tr>
<tr><td>P1</td><td>GCGAAAGCTAAGGATTTTTTTAAGCTTGGCACTGGCCGTCGT</td><td rowspan="4">pNEG-GFP190TAG-2chPheT</td></tr>
<tr><td>P2</td><td>TAAAAAAAATCCTTAGCTTTCGCTAAGGA</td></tr>
<tr><td>P3</td><td>ACTAGTCCCATCAAAAAAATATTCTCAACATAAAAAACTT</td></tr>
<tr><td>P4</td><td>ATTTTTTTGATGGGACTAGTTAAAAAAAATCCTTAGCTTTCGCTAAGGATCTGCAGTG</td></tr>
<tr><td>P5</td><td>CATTTATTCTGCCTCCCAGAGCATGATAAAAACG</td><td rowspan="2">pChira-oxb20-chPheRS-2*chPheT</td></tr>
<tr><td>P6</td><td>GGCAGAATAAATGGCTGAGTTGAAGGATCCTCGGGTTG</td></tr>
</table>

P7	CGATTCAGGTTCATCATGCCGTTTGTGATGG	
P8	GATGAACCTGAATCGCGAATTTGCTTTCGAATTTCTGCC	
P9	GAGCGCATTGTTAGCTGACTTCAGGTGCTACATTTGAAG	
P10	CTAACAATGCGCTCATCGTCATCCTCGGCAC	
P11	CGAGCCGATGATTAATTGTCAAGCCCGGCAGTACCGGCATAACCAAG	pChira-tac-oxb20-chPheRS-2*chPheT
P12	CAATTAATCATCGGCTCGTATAATGCATACCCGTTTTTTTGGGCTAG	
P13	ATGTATATCTCCTTCTTAAAGTTAAACAAAATTATTTCTAGC	pChira-tac-2*TAG-dnaN-oxb20-chPheRS-2*chPheT
P14	GTAGGTTCCAGGTTAGGGTGGTAAATTTACCGTAGAACGTGAG	
P15	GATGGTGATGATGCAGTCTCATTGGCATGACAACATAAG	
P16	CATCATCACCATCACCATTAAGCATGCACC	
P17	GTAGGTTTAGGGTTAGGGTGGTAAATTTACCGTAGAACGTGAG	pChira-tac-3*TAG-dnaN-oxb20-chPheRS-2*chPheT
P18	GTAGGTTTAGGGTTAGGGTTAGAAATTTACCGTAGAACGTGAG	pChira-tac-4*TAG-dnaN-oxb20-chPheRS-2*chPheT
P19	GAGATATACATATGTAGGTTTAGGGTTAGGGTGGTCGTATCATTCTGCTTGGCGCTC	pChira-tac-3*TAG-adk-oxb20-chPheRS-2*chPheT
P20	GATGGTGATGATGGCCGAGGATTTTTCCAGATC	
P21	GAGATATACATATGTAGGTTTAGGGTTAGGGTGGTCAATTTAATATCCCTACGTTGC	

P22	GATGGTGATGATGCTGATCAAGCAAATCTGCACG	pChira-tac-3*TAG-pgsA-oxb20-chPheRS-2*chPheT
P23	GAGATATACATATGTAGGTTTAGGGTTAGGGTGGTGCAAGCAGTAACTTGATTAAAC	pChira-tac-3*TAG-TyrS-oxb20-chPheRS-2*chPheT
P24	GATGGTGATGATGTTTCCAGCAAATCAGACAGTAATTC	
P25	CGCATCAGGCGCTCTTCCGCTTCCTCGCTC	pGEX-trp-fLuc
P26	GAGCGCCTGATGCGGAACTTTTGCTGAGTTGAAGG	
P27	TTTGGCATCTTCCATTTGCGACCTCCTTATGAAAGC	
P28	ATGGAAGATGCCAAAAACATTAAGAAGGGC	
P29	CAGTCAGTTAATGGTGATGGTGATGATGCACGGCGATC	
P30	CCATTAACTGACTGACGATCTGCCTCGCG	
P31	ATTGTCATCGTAAACCTGAAGTTCCTATACTTTCTAGAGAATAGGAACTTCGGAATAGG	Upstream$_{100bp}$-*ΔdnaN::KanR*-Dowmstream$_{100bp}$
P32	CAAATTTAATCAGAACATTGTCATCGTAAACCTGAAGTTCCTATACTTTCT	
P33	GAGCCACGATATCAAAGAAGATTTTTCAAATTTAATCAGAACATTGTCATCGTAAACCT	
P34	GTGCTTCATGCCTGCCGTAAGATCGAGCAGTTGCGTGAAGAGAGCCACGATATCAAAGAAGATTTTTCA	
P35	CAACAAGCGGGTGAGGGACAGAAGTTCCTATTCTCTAGAAAGTATAGGAACTTCAGAGC	
P36	AAATCGCGGATCAACAAGCGGGTGAGGGACAGA	

P37	CGGGAGATAAGGCGAGATCCGCGGTTTCAATGTTGCGGAAATCGCGGATCAACAAGCGG	
P38	CTGCCGTTGGCACCTACCAGAAAGTTAAAGCCGGGAGATAAGGCGAGATCCG	
P39	GATAGTTACCCGTCATTGAAGTTCCTATACTTTCTAGAGAATAGGAACTTCGGAATAGG	Upstream100bp-△*pgsA::KanR* -Dowmstream100bp
P40	CTGTAGCAACATAGGGGTAATCTTACTGACAACAGATAGTTACCCGTCATTGAAGTTCC	
P41	CGCAAGGTCTGGCAGAAAAGATCTTCTGGTCGTTGAAACATTGATGTCTCTGTAGCAACATAGGGGTAATCTTAC	
P42	TGAAAATTACGCCGAAACGAGAAGTTCCTATTCTCTAGAAAGTATAGGAACTTCAGAGC	
P43	AGTCAACGATATTTTTCACCACTTTTGATCGTTTGCTGAAAATTACGCCGAAACGAGAA	
P44	GTGCCGCCGTTCGATGCGTTGCATTCTACTTACCTGGCGCGATGAGTCAACGATATTTTTCACCACTTTTG	
P45	TTAAGGGGATTTTCGCAGAAGTTCCTATACTTTCTAGAGAATAGGAACTTCGGAATAGG	Upstream100bp-△*adk::KanR* -Dowmstream100bp
P46	GCTTTTTCAAAAAATTCGACACATTTTAAGGGGATTTTCGCAGAAGTTCCTATACTTTC	
P47	CCGTTTCAGCCCCAGGTGCCTTTCTTGAGGCAATCGCCTGTTGGTGGTATCGTTTATCGCTTTTTCAAAAAATTCGACACATTTTAAGG	
P48	TGTCCGTGCGCGCTTTCGAAGAAGTTCCTATTCTCTAGAAAGTATAGGAACTTCAGAGC	
P49	CCCCTCACCCTAACCCTCTCCCCGAGGGGGCGAGGGGACTGTCCGTGCGCGCTTTCGAA	

P50	CGGCCTGAGATTGCTGATAAGTTTGCTTGTGCGGGCCTGTTCCCCTCACCCTAACCCTCTC	
P51	TATACATGGAGATTTTGGAAGTTCCTATACTTTCTAGAGAATAGGAACTTCGGAATAGG	Upstream$_{100bp}$-△*TyrS::KanR*-Dowmstream$_{100bp}$
P52	GTCGTGTACCGGCAAAGGTGCAGTCGTTATATACATGGAGATTTTGGAAGTTCCTATAC	
P53	TGGTACTCCTGATTCTGGCACTTTATTCTATGTCTCTTTCGCATCTGGCGAAAAGTCGTGTACCGGCAAAGGTG	
P54	CCCCCTTTCCACTTAATGCAGAAGTTCCTATTCTCTAGAAAGTATAGGAACTTCAGAGC	
P55	TTCCCTGTATAAAAACCAGGGGGAGTGATTTCTCACTCCCCCTTTCCACTTAATGCAGA	
P56	ATAAACAACGTGAGACTGGATAGCGAGAATATTTTTCATCATGTTCCCTGTATAAAAACCAGGGGGAG	
P57	GTGCTTCATGCCTGCCGTAAGAT	Colony PCR of Upstream$_{100bp}$-△*dnaN::KanR*-Dowmstream$_{100bp}$
P58	CTGCCGTTGGCACCTACCAGAAAGT	
P59	AAGGTCTGGCAGAAAAGATCTTCT	Colony PCR of Upstream$_{100bp}$-△*pgsA::KanR*-Dowmstream$_{100bp}$
P60	GTTCGATGCGTTGCATTCTACT	
P61	CCGTTTCAGCCCCAGGTGC	Colony PCR of Upstream$_{100bp}$-△*adk::KanR*-Dowmstream$_{100bp}$
P62	CGGCCTGAGATTGCTGATAAGT	
P63	TGGTACTCCTGATTCTGGCACTT	Colony PCR of Upstream$_{100bp}$-
P64	ATAAACAACGTGAGACTGGATAGCGA	

		△*tyrS::KanR*-Dowmstream$_{100bp}$
P65	GGAGATATACATATGGCCAACATCGCGGTGCAGCG	pNEG-UBE2K-2*chPheT
P66	GATGGTGATGATGGTTACTCAGAAGCAATTCTGTTG	
P67	GAGATATACATATGCGTATCATTCTGCTTGGCGC	pNEG-ADK-2*chPheT
P68	GATGGTGATGATGGCCGAGGATTTTTTCCAGATCAG	
P69	GAGATATACATATGGCAAGCAGTAACTTGATTAAAC	pNEG-TyrRS-2*chPheT
P70	GATGGTGATGATGTTTCCAGCAAATCAGACAGT	
P71	GGAGATATACATATGGAAGATGCCAAAAACATTAAG	pNEG-Fluc-2*chPheT
P72	GATGGTGATGATGCACGGCGATCTTGCCGCCCTTC	
P73	GATCAGCTAGTCCGGTACGTCGAATTCCAG	pNEG-ADK-E114TAG-2*chPheT
P74	CGTACCGGACTAGCTGATCGTTGACCGTATCGTCG	
P75	GCCTTCCTATTTCGGCGGATTGAATTTAACGTG	pNEG-ADK-V142TAG-2*chPheT
P76	CCGCCGAAATAGGAAGGCAAAGACGACGTTAC	
P77	GTGTCTATGGAGGTCCTGCTATTTCTCCTCTTAATTC	pNEG-UBE2K-D48TAG-2*chPheT
P78	GCAGGACCTCCATAGACACCATATGAAGGAGGAAG	
P79	GTTTCTACTGATTTGCTACTACAGCATCCTGTGGATC	pNEG-UBE2K-Y134TAG-2*chPheT

P80	GTAGCAAATCAGTAGAAACAAAATCCCGAAATGTTC	
P81	GGTCTAGGGGATGATCTGGTTGCCGAAGATGGGG	pNEG-Fluc-D234TAG-2*chPheT
P82	CAGATCATCCCCTAGACCGCTATCCTCAGCGTGG	
P83	GCGCTACATGAGCACGACCCGAAAGCCGCAGATC	pNEG-Fluc-Y266TAG-2*chPheT
P84	GTCGTGCTCATGTAGCGCTTCGAGGAGGAGCTATTC	
P85	CCCTAAGTTTTACCAAATTTGGTGCCATCTGCTTTAG	pNEG-TyrRS-E240TAG-2*chPheT
P86	TGGTAAAACTTAGGGCGGCGCAGTCTGGTTGGATC	
P87	GTACCTACTGGGCGCGCGGTGCTTTACCGCTG	pNEG-TyrRS-Y231TAG-2*chPheT
P88	GCGCGCCCAGTAGGTACTGGCGGAGCAGGTGAC	
P89	GACTACAACATCCAGAAAGAGTCCA	pNEG-Ub-S57TAG-2*chPheT
P90	TTTCTGGATGTTGTAGTCCTACAGGGTACGACCATCTTCCA	
P91	ACAACATCCAGAAAGAGTAGACCTTGCACCTGGTGCTCCG	pNEG-Ub-S65TAG-2*chPheT
P92	CTCTTTCTGGATGTTGTAGTCAGACAG	
P93	CTTGGAGCCGCTGTAGAGCAGG	pNEG-GID4(116-300)-F128TAG-2*chPheT
P94	TGCTCTACAGCGGCTCCAAGTAGCGCGGCCACCAGAAGAGCAA	
P95	GATGCACTGATGATCCTGAATATCGT	pNEG-Neo2/15-Y14TAG-2*chPheT
P96	ATTCAGGATCATCAGTGCATCCTACAGTGCATGTTCAGCGTGCA	
P97	CAGTTTTGTATCCATATGTATATCTCCTTCTT	

P98	TACATATGGATACAAAACTGTAGTGCATCTG TAAAACGCCGTATG	pNEG-BPTF (2583-2751)-GST-Y2587TAG
P99	TATCATGGTCGTTGTGTGGGTATTCTG	pNEG-BPTF (2583-2751)-GST-W2609TAG
P100	CCCACACAACGACCATGATACTAATTCTGA CAACGATCGCAACCAATAT	
P101	TAAGTTGGGTAACGCCAGGGAGTAAGAGG CACTCTACATGTG	pChira-oxb20-chPheRS-2*chPheT-CmR-Site1
P102	CCCTGGCGTTACCCAACTTAAT	
P103	TTTTCCCAGTCACGACGTTGTAA	
P104	CAACGTCGTGACTGGGAAAATGCTTGTTAG GCCAATATTTCAAA	
P105	TGATCTTCCGTCACAGGTATTTATTCG	
P106	ATACCTGTGACGGAAGATCATCCCGAGGCA TCACTGCTAAA	
P107	GATTTATTTATTCTGCGAAGTCGCCGACTGA CAACAAATTAATTA	
P108	CTTCGCAGAATAAATAAATCCTGGTGTC	
P109	AACAACTTTGCAGATTAATTAACCAATTG	pChira-oxb20-chPheRS-2*chPheT-KanR-Site2
P110	CAACGTCGTGACTGGGAAAACATAGCAAA ACGCCCTCAAC	
P111	ATACCTGTGACGGAAGATCAGCAAGTTGA GTTATCAGACAGATACCC	
P112	GATTTATTTATTCTGCGAAGAACGCCATCAC CATTTTCCC	
P113	TCAAGTGTTTTGTAGAAATTGTTGCCACAA GGTCTGTTATAAGCGGGACAGGCTGACAA	pBK-oxb15-12D4-AzFRS-2-chPheT
P114	GTTGAAGCAATTATACTGTTTTTTTTACAG TATCAAGTGTTTTGTAGAAATTGTTGCC	
P115	AACAGTATAATTGCTTCAACAGAACATATT GACTATCCGGTATTACCCGGCATGACAGG	

P116	GGTATTACCCGGCATGACAGGAGTAAAAAT GGCTATCCGCCGTTATACGTTGTTTACGC	
P117	AGTAAGAGGCACTCTACATGTGTTC	OTS integration cassette 1
P118	TCCCGAGGCATCACTGCTAAA	
P119	AACAACTTTGCAGATTAATTAACCAATTG	OTS integration cassette 2
P120	GCAAGTTGAGTTATCAGACAGATAC	

References

1. Chin JW, Santoro SW, Martin AB, King DS, Wang L, Schultz PG. Addition of p-azido-L-phenylaianine to the genetic code of Escherichia coli. *J Am Chem Soc* 2002, **124**(31)**:** 9026-9027.

2. Amiram M, Haimovich AD, Fan C, Wang Y-S, Aerni H-R, Ntai I*, et al.* Evolution of translation machinery in recoded bacteria enables multi-site incorporation of nonstandard amino acids. *Nat Biotechnol* 2015, **33**(12)**:** 1272-1279.
