# Supplementary material for: Directed-evolution of translation system for efficient unnatural amino acids incorporation and generalizable synthetic auxotroph construction
Source: Nat Commun. 2021 Dec 2;12:7039. doi: 10.1038/s41467-021-27399-x (PMC8639764; doi:10.1038/s41467-021-27399-x)
Supplement: Supplementary file 2 — Reporting Summary [file 41467_2021_27399_MOESM2_ESM.pdf]

nature research

Corresponding author(s): Shixian Lin

Last updated by author(s): Nov 15, 2021

Reporting Summary

Nature Research wishes to improve the reproducibility of the work that we publish. This form provides structure for consistency and transparency in reporting. For further information on Nature Research policies, see our Editorial Policies and the Editorial Policy Checklist.

Statistics

For all statistical analyses, confirm that the following items are present in the figure legend, table legend, main text, or Methods section.

n/a	Confirmed	
☐	☒	The exact sample size (*n*) for each experimental group/condition, given as a discrete number and unit of measurement
☐	☒	A statement on whether measurements were taken from distinct samples or whether the same sample was measured repeatedly
☐	☒	The statistical test(s) used AND whether they are one- or two-sided<br>*Only common tests should be described solely by name; describe more complex techniques in the Methods section.*
☒	☐	A description of all covariates tested
☒	☐	A description of any assumptions or corrections, such as tests of normality and adjustment for multiple comparisons
☐	☒	A full description of the statistical parameters including central tendency (e.g. means) or other basic estimates (e.g. regression coefficient) AND variation (e.g. standard deviation) or associated estimates of uncertainty (e.g. confidence intervals)
☐	☒	For null hypothesis testing, the test statistic (e.g. *F*, *t*, *r*) with confidence intervals, effect sizes, degrees of freedom and *P* value noted<br>*Give P values as exact values whenever suitable.*
☒	☐	For Bayesian analysis, information on the choice of priors and Markov chain Monte Carlo settings
☒	☐	For hierarchical and complex designs, identification of the appropriate level for tests and full reporting of outcomes
☒	☐	Estimates of effect sizes (e.g. Cohen's *d*, Pearson's *r*), indicating how they were calculated

Our web collection on statistics for biologists contains articles on many of the points above.

Software and code

Policy information about availability of computer code

Data collection	GFP fluorescence for assessment of amber suppression efficiency were collected with Gen5 CHS 2.09 software. Growth curve of DH10B strains were collected with Gen5 CHS 2.09 software. Bioluminescence signals for monitoring the survival of UAA-dependent synthetic auxotroph in presence or absence of UAA were collected with Gen5 CHS 2.09 software in vitro, with IVIS Spectrum in living mouse. Growth curve of UAA-dependent synthetic auxotrophs were collected with spectrophotometer DU730. FACS of HEK 293T cells were acquired with CytExpert 2.0. Western blotting PVDF membranes were captured by cSeries Capture Software.
Data analysis	The assessment of activity for chimeric pairs were processed with Origin 8.0 software. Comparisons of activity for UAA incorporation with different orthogonal pairs were performed using one-way ANOVA with significance level at $\alpha = 0.05$ in Origin 8.0 software. The growth curve of strains were processed with Origin 8.0 software. Mass spectral deconvolution was performed using UNIFI software (version 1.9.4, Waters Corporation). FACS data were processed with FlowJo (version 10, Treestar Software).

For manuscripts utilizing custom algorithms or software that are central to the research but not yet described in published literature, software must be made available to editors and reviewers. We strongly encourage code deposition in a community repository (e.g. GitHub). See the Nature Research guidelines for submitting code & software for further information.

Data

Policy information about availability of data

All manuscripts must include a data availability statement. This statement should provide the following information, where applicable:

- Accession codes, unique identifiers, or web links for publicly available datasets
- A list of figures that have associated raw data
- A description of any restrictions on data availability

Any Supplementary Information (methods, figures, DNA sequences, and protein sequences) and chemical compound information are available in the online version

of the paper. The source data for Figure 2D-E, 3A, 3C-E, 4A-D, 5A, 5C-E, and 6A-C, and Supplementary Figure 4, 5, 6, 7, 8, 9, 10, 11, 12, 13, and 14 are provided with this paper as a Source Data file. Correspondence and requests for materials should be addressed to S.L.

Field-specific reporting

Please select the one below that is the best fit for your research. If you are not sure, read the appropriate sections before making your selection.

[x] Life sciences   [ ] Behavioural & social sciences   [ ] Ecological, evolutionary & environmental sciences

For a reference copy of the document with all sections, see nature.com/documents/nr-reporting-summary-flat.pdf

Life sciences study design

All studies must disclose on these points even when the disclosure is negative.

Sample size	Sample size was not predetermined and was empirically set at n=3 independent biological replicates. The sample size for each experiment is also indicated in the figure legend.
Data exclusions	No data were excluded in this study.
Replication	All experiments were repeated independently at least twice with similar results. At least three different biological replicates were performed for quantitative experiments.
Randomization	E. coli, mammalian cells and mouse used in this study were randomly assigned as controls or treatment groups. Single clones from plate for assessment of amber suppression efficiency were randomly picked.
Blinding	The investigators collecting and analyzing data like imaging, fluorescent intensity data in this study were blind to the experimental conditions. For other experiment, the investigators were not blinded to sample identity, since the data was from objective quantitative methods so subjective bias was not relevant.

Reporting for specific materials, systems and methods

We require information from authors about some types of materials, experimental systems and methods used in many studies. Here, indicate whether each material, system or method listed is relevant to your study. If you are not sure if a list item applies to your research, read the appropriate section before selecting a response.

Materials & experimental systems

n/a	Involved in the study	
[ ]	[x]	Antibodies
[ ]	[x]	Eukaryotic cell lines
[x]	[ ]	Palaeontology and archaeology
[ ]	[x]	Animals and other organisms
[x]	[ ]	Human research participants
[x]	[ ]	Clinical data
[x]	[ ]	Dual use research of concern

Methods

n/a	Involved in the study	
[x]	[ ]	ChIP-seq
[ ]	[x]	Flow cytometry
[x]	[ ]	MRI-based neuroimaging

Antibodies

Antibodies used	Primary antibody: Anti-GFP rabbit antibody (Cell Signaling Technology, cat#2555, lot#6) was used in a dilution of 1:1000; Secondary antibody: Goat anti-rabbit IgG (H+L), HRP conjugate (Proteintech, cat#SA00001-2, lot#20000311) was used in a dilution of 1:5000.
Validation	Antibodies were validated by manufacturer and statements were found in the following links .<br>Anti-GFP rabbit antibody was cited by 179 papers based on the information on the website (https://www.cellsignal.com/products/primary-antibodies/gfp-antibody/2555)<br>Goat anti-rabbit IgG (H+L) was cited by 3647 papers based on the information on the website (https://www.ptglab.com/products/HRP-conjugated-Affinipure-Goat-Anti-Rabbit-IgG-H-L-secondary-antibody.htm#product-information)

Eukaryotic cell lines

Policy information about cell lines

Cell line source(s)	HEK 293T cell is from ATCC

Authentication	The cell line was used to test protein expression only, so the cell line is not authentication.
Mycoplasma contamination	The HEK 293T cell line tested negative for mycoplasma contamination.
Commonly misidentified lines (See ICLAC register)	No commonly misidentified lines used in this study.

Animals and other organisms

Policy information about studies involving animals; ARRIVE guidelines recommended for reporting animal research

Laboratory animals	Balb/c mice (male, 6-8 weeks) were purchased from Shanghai Model Organisms. All mice were reared in-house (temperature: 20–25°C,humidity: 40%–60%) in 12h light/dark cycle. All animals had free access to food and sterilized water.
Wild animals	This study did not involve wild animals.
Field-collected samples	This study did not involve field-collected samples.
Ethics oversight	Care of experimental animals was in accordance with guidelines and approved by the Laboratory Animal Committee of Zhejiang University.

Note that full information on the approval of the study protocol must also be provided in the manuscript.

Flow Cytometry

Plots

Confirm that:

- [x] The axis labels state the marker and fluorochrome used (e.g. CD4-FITC).
- [x] The axis scales are clearly visible. Include numbers along axes only for bottom left plot of group (a 'group' is an analysis of identical markers).
- [x] All plots are contour plots with outliers or pseudocolor plots.
- [x] A numerical value for number of cells or percentage (with statistics) is provided.

Methodology

Sample preparation	FACS analysis of live cells, HEK 293T cells were grown in 6-well plates (Corning) and co-transfected with the pcDNA3.1 vector bearing the aaRS/tRNA pair and pEGFP-mCherry-T2A-GFP190TAG-His6 at ratio 1:1 (µg: µg). Transfection was performed using lip2000 reagent (BioSharp) according to the manufacture's protocol with or without the addition of the corresponding UAA at the final concentration of 2 mM. After transfection (48h), cells were trypsinized and taken up in full medium before centrifugation. Cells were centrifuged at 1400 g for 3 min, washed, and resuspended in PBS.
Instrument	Beckman CytoFlex
Software	FlowJo
Cell population abundance	At least 50000 single cells were analyzed per condition. GFP fluorescence was acquired at the FITC channel, and mCherry fluorescence was acquired at the PE channel. Purity of 293T cells is 99% or higher.
Gating strategy	HEK 293T cells were used to set appropriate forward scatter (FSC) and side scatter (SSC) gains. The GFP protein expressed cells were used to set FITC gains and gate and the mCherry protein expressed cells were used to set PE gains and gate.

- [x] Tick this box to confirm that a figure exemplifying the gating strategy is provided in the Supplementary Information.
